# Modeling the impact of antiretroviral therapy on HIV and related kidney diseases in Tanzania

**DOI:** 10.1038/s41598-025-94114-x

**Published:** 2025-03-17

**Authors:** Janeth Pancras Mchwampaka, Miracle Amadi, Nyimvua Shaban Mbare

**Affiliations:** 1 Department of mathematics, University of Dar es Salaam, Dar es Salaam, Tanzania; 2 LUT School of Engineering Sciences, Lappeenranta-Lahti University of Technology (LUT), Yliopistonkatu 34, Lappeenranta, Finland

**Keywords:** Mathematical model, HIV-related kidney diseases, Reproduction number, Sensitivity analysis, Markov chain Monte Carlo, Computational biology and bioinformatics, Mathematics and computing

## Abstract

This work presents a mathematical model for the dynamics of HIV-related kidney diseases. The study examines two cases, considering the effects of the absence of treatment and the effects of Highly Active Antiretroviral Therapy (HAART) on different infection groups. Studying these cases is important because many developing countries implement HAART late, and not all HIV-infected individuals receive this therapy. Kidney diseases in HIV individuals are modeled as arising from both HIV infection itself and the use of nephrotoxic drugs. In the analysis of the mathematical model, it is shown that the state variables of the model are non-negative and bounded. Furthermore, we derived a formula for control reproduction number $R_c$ which was used to compare the cases considered. The sensitivity analysis of the model reveals that the effect of HAART in reducing the progression from HIV to HIV-related kidney diseases is more significant compared to other effects of HAART on disease dynamics, which is also confirmed through numerical simulations. The Markov Chain Monte Carlo (MCMC) method was used to estimate parameters and evaluate the model using real data of the HIV population from Tanzania from 1990 to 2022. Numerical simulations demonstrated that while HAART reduces HIV progression to the AIDS stage, the population of individuals with HIV-related kidney diseases is increasing and is projected to continue increasing. Additionally, the results show that improving the effectiveness of HAART by 90% in preventing the progression from HIV to HIV-related kidney diseases can significantly decrease the prevalence of HIV-related kidney diseases. This study addresses a gap in understanding how population-level HAART availability influences the dynamics of HIV-related kidney disease, particularly in settings with delayed or inconsistent treatment access. By analyzing disease progression under these conditions, our findings provide insights that can inform public health strategies for improving HIV care in resource-limited settings and other contexts where access disparities persist. In addition, the work investigated scenarios related to data quality in which the model parameters can be well identified, which can serve as a guide for obtaining informative real data.

## Introduction

The retrovirus known as human immunodeficiency virus (HIV) affects the human immune system by encoding its genetic material as ribonucleic acid (RNA)^1,2^. According to^3^, 37.7 million people were living with HIV/AIDS and there were 680, 000 deaths from AIDS-related diseases worldwide in 2020. Since the introduction of highly active antiretroviral therapy (HAART) in 1996, there has been significant improvement in the morbidity and mortality of HIV-infected patients^4^. However, the increased accessibility to HAART has led to new challenges, including a higher burden of renal and genitourinary diseases among HIV-infected individuals. These diseases can be caused directly by HIV, indirectly by opportunistic infections, HAART-related toxicities (nephrotoxicity), and non-communicable diseases such as obesity, hypertension, and diabetes^5^.

Chronic kidney disease (CKD) is a condition where the kidneys are damaged and cannot adequately filter blood. Risk factors for kidney disease include diabetes, high blood pressure, and heart failure^6^. In HIV-infected individuals, CKD and HIV-associated nephropathy (HIVAN) are non-infectious complications that can progress to end-stage renal disease (ESRD)^7,8^. The relationship between HIV and kidney disease was first reported in New York and Miami in 1984^9^. However, research on kidney diseases in HIV/AIDS-infected individuals is limited in middle and low-income countries.

Tanzania, like many other Sub-Saharan countries, faces a growing burden of non-communicable diseases such as cardiovascular diseases, diabetes, and kidney dysfunction. These non-communicable diseases have strained the health system, which was initially overwhelmed by managing contagious diseases (Mayige et al. 2012). Kidney diseases have significantly contributed to the increased risk and burden of cardiovascular problems as well as mortality in Tanzania and other low- and middle-income countries^10^. Multiple studies^11–13^ conducted in Tanzania have consistently reported a high prevalence of CKD among HIV patients receiving HAART, with factors such as advanced age, male gender, hypertension, and high viral load associated with CKD^11,13^. For instance^12^, demonstrated a high prevalence of kidney dysfunction in adult patients with associated mortalities in Mwanza, Tanzania.

Another study by Peck et al.^14^ showed that HIV-infected adults in Tanzania who have been on antiretroviral therapy (ART) for more than two years have a high risk of developing hypertension and kidney disease. This was supported by Mapesi et al.^11^ who conducted research in rural Tanzania, who demonstrated the prevalence of renal impairment among people living with HIV with high usage of ART. Despite the widespread use of antiretroviral treatment reducing the incidence of opportunistic infections among HIV-infected patients and improving health and life expectancy to near normal, kidney disease remains a significant health problem among individuals with HIV in Tanzania.

Based on research conducted in Tanzania, kidney disease is a significant health problem among individuals with HIV across all sub-populations. According to^15^, the prevalence of renal dysfunction increases by $3\%$ for each year of age. Additionally^16^, found that as age increases, the rate of kidney disease also increases among HIV-infected individuals. Furthermore, the duration of time on HAART is identified as a predictor of kidney diseases among HIV individuals. Since the prevalence of HIV/AIDS in the general population is $4.7\%$ and individuals in Tanzania tend to start HAART early, the burden of kidney diseases among individuals with HIV is likely significant^17^.

The study of^18^ found that Adults on HAART were more likely to have kidney diseases and using the HAART combination of Tenofovir, Lamivudine, and Efavirenz caused kidney damage. However, other HAART combinations did not harm the kidneys. The findings suggest that kidney disease in HIV/AIDS individuals can be linked to certain types of HAART treatments. The study of^19^ evaluated the impact of highly active antiretroviral therapy (HAART) on renal function among HIV-infected Ugandans in the Home-Based AIDS Care clinical trial. Their study considered patients with symptomatic HIV disease or CD4 cell count $< or = 250 \;{\text{cells}}/{\text{mm}}^{3}$ and the result shows that renal dysfunction was common with advanced HIV disease in Uganda but this improved following 2 years of HAART.

According to^20^ age greater than 50 years, male gender, hypertension, diabetes mellitus, high triglycerides nadir, CD4 cell count$<200 \; {\text{cells}}/{\text{mm}}^{3}$, current use of tenofovir diisopropyl fumarate (TDF) and of TDF plus ritonavir-boosted protease inhibitors were independently associated with CKD, while current use of abacavir plus one integrase inhibitor was associated with a reduced risk of CKD. Also^21^, study showed that high blood pressure and advanced AIDS stages were significantly associated with chronic kidney disease in HIV patients, whether they had started HIV treatment(HAART) or not. A cohort study by Mocroft et al.^22^ found that for each additional year of taking certain HIV medications such as tenofovir disoproxil fumarate, atazanavir boosted with ritonavir and lopinavir boosted with ritonavir, there was a significant increase in chronic kidney disease. However, other similar medications or abacavir did not show this association with kidney disease risk and their study had a similar result as studies of^23,24^. Studies have shown that HAART lowers the risk of kidney disease in HIV patients, but other treatments can lead to kidney issues. This study did not focus on the specific HAART drugs that were used; it only looked at the overall impact of HAART on HIV patients.

Due to the common occurrence of HIV-related kidney diseases in African populations and the current practice of initiating HAART regardless of CD4 cell counts, there has been a noticeable increase in kidney disease prevalence. Mathematical models have been used to study the impact of HAART on HIV and HIV-related kidney diseases^8,25,26^. The study of^8^ developed a mathematical model that highlighted the importance of reducing AIDS cases to lower the prevalence of HIV-related kidney diseases. They found that HAART’s effectiveness in preventing AIDS and reducing mortality influences the reduction of the prevalence of kidney diseases over time. Also, the study of^25^ uses an ordinary differential equation model to explore the impact of antiretroviral therapy (ART) on HIV-associated end-stage renal disease (ESRD) dynamics among African Americans. Simulations show that ART reduces ESRD prevalence initially, but levels rise again unless ART coverage is near $100\%$. Their findings highlight the critical need for high ART effectiveness to mitigate long-term HIV/ESRD outcomes.

This study investigates the relationship between HAART and HIV-related kidney diseases in Tanzania, using data from the United Nations Programme on HIV/AIDS (UNAIDS). Rather than differentiating between specific HAART regimens, the model examines the overall impact of HAART accessibility and implementation on disease progression at the population level. Two scenarios are considered: one where HAART is absent and another where it is implemented, reflecting real-world challenges where HAART initiation may be delayed or unequally distributed across patient groups. To estimate model parameters and quantify uncertainty, this study employs Markov Chain Monte Carlo (MCMC) methods, a standard approach for parameter estimation. MCMC efficiently samples from complex probability distributions that are intractable with analytical techniques. It is particularly well-suited for high-dimensional problems with intricate correlations, providing full posterior distributions that capture both parameter uncertainty and interdependencies^27^. Despite its computational demands, MCMC is highly effective in handling noisy or sparse datasets where traditional resampling approaches may struggle^28^. The method has been widely applied in estimating parameters for complex systems, as demonstrated in previous studies^26,29,30^. Additionally, this study introduces a structured approach to parameter identification across varying data availability scenarios, offering a methodological framework that can guide the collection of informative real-world data.

## Model description

According to the Centers for Disease Control and Prevention (CDC), HIV has three stages: acute HIV, chronic HIV, and advanced HIV/full-blown AIDS^31^. A deterministic model is formulated in which the human population is divided into four compartments to describe how individuals progress from one compartment to another: susceptible (*S*(*t*)) are individuals who are free from HIV infection but are capable of becoming infected. HIV compartment (*H*(*t*)) includes all individuals who have been infected with HIV but CD4 cells are greater than $200 \; {\text{cell}}/{\text{mm}}^{3}$ and transmit the disease to others at the rate $(k_2)$. As the body’s immunity weakens a certain proportion of individuals from the HIV compartment progress to the AIDS compartment at rate $\rho$. Individuals in HIV/AIDS-related kidney diseases compartment (*K*(*t*)) are HIV-infected and can have kidney diseases either before or after entering stage, 3 considering that kidney disease in HIV/AIDS individuals results from co-infection, long-term HIV infection, and treatment. This compartment includes HIV and AIDS individuals who progress to *K*(*t*) at the rates of $s_1$ and *s* respectively. To make the model less complex and simple to be mathematically analyzed, the study considers the following assumptions: In susceptible and HIV compartments, individuals die naturally at rate $\mu$, and in AIDS and HIV-related kidney diseases compartment individuals die due to a given disease with rate $\mu _{A}$ and $\mu _{K}$ respectively.

Due to the use of HAART, the life expectancy of HIV-infected patients has increased, and now most deaths result from non-AIDS-related complications rather than AIDS-defining illnesses^32^, thus, $k_7$ represents the effect of HAART to reduce the mortality due to AIDS and $k_3$ represents the effect of HAART to reduce the progression from HIV without symptom to AIDS.

Also, kidney disease caused by HAART is much more common than HIV-associated nephropathy (HIVAN). Patients with HIVAN usually have a CD4 count below 200 cells/mm, while those with HAART-related kidney disease have a CD4 count above 200 cells/mm. It is important to tell the difference between HIVAN and HAART-induced kidney disease because they need different treatments. The use of Angiotensin-converting enzyme inhibitor to block the renin-angiotensin-aldosterone system (RAAS) helps improve kidney health and should be added to HAART in cases of HIVAN. If kidney problems continue despite HAART and RAAS treatment, steroids can be added as an additional treatment^33^. Therefore, HAART helps slow down the progression from AIDS to HIV/AIDS-related kidney diseases, as shown by $k_4$ also $k_6$ represents the effect of HAART to reduced mortality due to kidney diseases. Additionally, $k_5$ shows that HAART reduces the progression from early-stage HIV (stages 1 and 2) to HIV/AIDS-related kidney diseases.

**Table 1:** Description of parameters and symbols used in the Model.

Parameter	Epidemiological description	Value	Source
$k_1$	Recruitment rate of susceptible	Estimated	
*s*	Progression rate from AIDS to HIV + kidney disease	0.01034	^8^
$s_1$	Progression rate from HIV to HIV + kidney disease	0.08759	^8^
$\rho$	Progression rate from HIV to AIDS	0.75998	^8^
$k_4$	Effect of HAART to reduce the progress from AIDS to HIV + kidney disease	Estimated	
$k_6$	Effect of HAART to reduce mortality due to HIV + kidney disease	Estimated	
*c*	Death rate due to AIDS	0.1	^25^
$k_7$	Effect of HAART to reduce mortality due to AIDS	Estimated	
$\mu$	Natural death rate	1/67.3	^34^
$k_2$	Effective transmission rate	Estimated	
$\delta$	Mortality rate due to HIV + kidney disease	0.67	^8^
$k_5$	Effect of HAART to block the progression from HIV to HIV + kidney disease	Estimated	
$k_3$	Effect of HAART to block the progression from HIV to AIDS	Estimated	
$\mu _{A}$	Natural mortality rate of AIDS population	1/11	^35^
$\mu _{K}$	Natural mortality rate of HIV + Kidney disease population	0.2	^8^

**Fig. 1:**
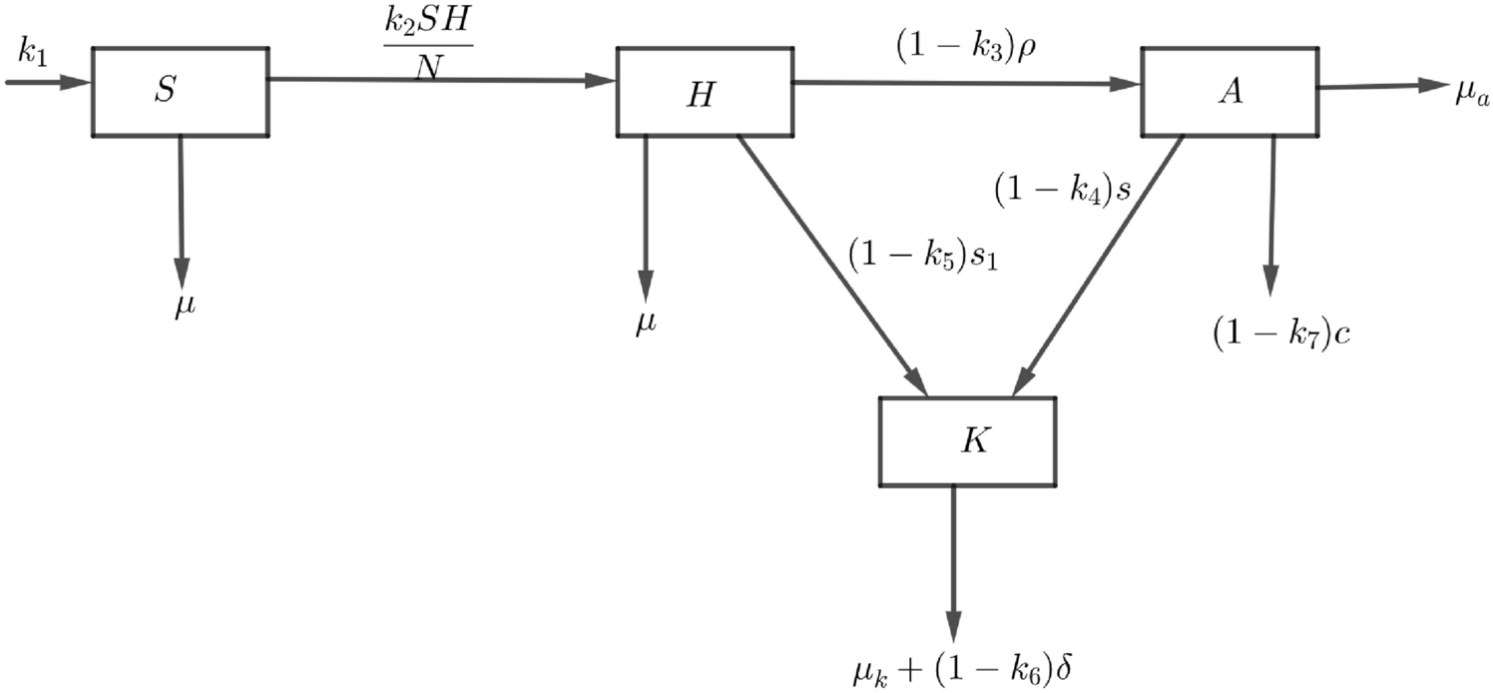
Flow diagram of HIV/AIDS-related kidney diseases.

From Fig. 1, and the model assumptions we get the following nonlinear system of differential equation;

1 $$\begin{aligned} \begin{aligned} \displaystyle {\dfrac{dS}{dt}}&=k_1 -\displaystyle {k_2 \frac{SH}{N}-\mu S},&\\ \displaystyle {\dfrac{dH}{dt}}&=\displaystyle {k_2 \frac{SH}{N}} -(1-k_3)\rho H-\mu H-(1-k_5)s_{1}H,&\\ \displaystyle {\dfrac{dA}{dt}}&=(1-k_3)\rho H-(1-k_7)cA-\mu _{A}A-(1-k_4)sA,&\\ \displaystyle {\dfrac{dK}{dt}}&=(1-k_4)sA+(1-k_5)s_{1}H-\mu _{K}K-(1-k_6)\delta K,&\end{aligned} \end{aligned}$$

with initial condition $S(0)>0,H(0)\ge 0,A(0)\ge 0,K(0)\ge 0$. The total population *N*(*t*) at any time (t) is given by $N(t)=S(t)+H(t)+A(t)+K(t)$. Table 1 provides a description of all parameters, including the values for the fixed parameters and their references.

##  Analysis of the model

The model system (1) represent the dynamic of HIV-related kidney diseases population in the presence of HIV treatment. For the model to be epidemiologically meaningfully, it is required to prove that all solution with initial conditions will remain positive and bounded at all time $(t\ge 0)$

### Positivity and boundness of the model

Since the system deals with a human population which required to be biologically valid and mathematical well-posed. it is important to show that all state variables are always non-negative with positive initial conditions in a bounded region. It is required to show that the solution of the model system (1) is non-negative and bounded for all time $t>0$. The following theorems are considered to prove the non-negativity and boundedness of the solution of model (1);

#### Non -negativity of the solutions

##### Theorem 1

*At initial conditions, the solutions of model system* (1) *are non-negative for all time* $t>0$.

##### Proof

Suppose that the initial condition of the state variables are non-negative, then first equation of the model (1) become,

$\dfrac{dS}{dt}=k_1 -\frac{k_2 SH}{N}-\mu S \ge -(\frac{k_2 H}{N}+\mu )S$. That is ,$\dfrac{dS}{dt}=\ge -(\frac{k_2 H}{N}+\mu )S$, implying that

$\displaystyle {S(t)\ge S(0) e^{-\int _{0}^{t}(\frac{k_2 H}{N}+\mu )dt} >0}$ as $t \Rightarrow \infty$ since the exponential function has non-negative quantity.

Similarly, other solutions of the model can be represented as

$$\displaystyle {H(t)\ge H(0)e ^{-\int _{0}^{t}((1-k_3)\rho +\mu +(1-k_5)s_{1})t}}>0$$,

$$\displaystyle {A(t)\ge A(0)e ^{-\int _{0}^{t}((1-k_7)cA+\mu _{A}+(1-k_4)s)dt}}>0$$,

$\displaystyle {K(t)\ge K(0)e ^{-\int _{0}^{t}(\mu _{K}+(1-k_6)\delta )dt}}>0$. As $t \Rightarrow \infty$, all the solutions approach initial condition.

Hence, all the solutions of the system (1) are non-negative for all $t>0$. $\square$

#### Biological feasible region

In mathematical modeling of infectious diseases, including HIV, the biologically feasible region refers to the subset of the state space where all model variables take on non-negative values and are bounded within realistic limits. This region ensures that the model remains meaningful in a biological and epidemiological context, preventing scenarios such as negative populations^36^.

##### Theorem 2

*All solutions of the system* (1) *are bounded in the region* $\Omega =\biggl \{ (S,H,A,K)\in \mathbb {R}_{+}^{4},N\le \frac{k_1}{\mu }\biggr \}$

##### Proof

Given that $N(t)=S(t)+H(t)+A(t)+K(t)$, by adding Eq. (1) yields

$$\displaystyle {\frac{dN}{dt}=k_1-\mu (S+H) -(1-k_3)\rho H-(1-k_7)cA-\mu _{A}A-\mu _{K}K-(1-k_6)\delta K}$$

$\displaystyle {\frac{dN}{dt} \le k_1-\mu N}$. By integration $\displaystyle {N(t)\le \left( N(0)-\frac{k_1}{\mu }\right) e^{-(\mu t)}+\frac{k_1}{\mu }}$, $\forall t\ge 0$ showing that $N(t)\longrightarrow \displaystyle {\frac{k_1}{\mu }}$ as $t \longrightarrow \infty$. Thus, the domain $\Omega$ is positive invariant.

Hence, the model is bounded and the system is epidemiological meaningful and well-posed. $\square$

### Existence of equilibrium solutions

#### Disease-free equilibrium point of the model

We analyze the model to determine the conditions necessary for the existence of an equilibrium/steady state which is referred to as the HIV-free kidney diseases steady state(disease-free equilibrium). This is the equilibrium at which the population remains in the absence of HIV infection. Therefore, to find the steady state we equate all terms containing HIV infection in Eq. (1) to be zero such as $H^{*}=A^{*}=K^{*}=0$. Then we have;

$$\begin{aligned} k_1 -k_2 SH-\mu S=0 \quad \Rightarrow S^{*}=\frac{k_1}{\mu } \end{aligned}$$

Therefore the free disease equilibrium can be represented as

$$\begin{aligned} E_{o}=(S^{*},H^{*},A^{*},K^{*})= \left( \dfrac{k_1}{\mu },0,0,0\right) . \end{aligned}$$

#### The control reproduction number

The definition of the control reproduction number $(R_c)$ in the context of our model is the number of secondary cases produced by HIV-infected individuals, in the presence of HIV treatment(HAART) as a control strategy^37^. The control reproduction number of the system was derived by using a next-generation method proposed by^38^. The matrices $\mathcal {F}_i$, representing the new infections, and $\mathcal {V}_i$ capturing all other transitions within the infectious stages of the model, are defined as follows;

$${\mathcal{F}}_{i} = \left[ {\begin{array}{*{20}c} {\frac{{k_{2} S}}{N}} \\ 0 \\ 0 \\ \end{array} } \right],\;{\mathcal{V}}_{i} = \left[ {\begin{array}{*{20}l} {(1 - k_{3} )\rho H + \mu H + (1 - k_{5} )s_{1} H} \hfill \\ { - (1 - k_{3} )\rho H + (1 - k_{7} )cA + \mu _{A} A + (1 - k_{4} )sA} \hfill \\ { - (1 - k_{6} )\delta K + \mu _{K} K - (1 - k_{5} )s_{1} A - (1 - k_{4} )sA} \hfill \\ \end{array} } \right].$$

The Jacobian matrix of $\mathcal {F}$ and $\mathcal {V}$ is determined by computing the partial derivatives of $\mathcal {F}_i$ and $\mathcal {V}_i$ with respect to the infectious groups *H*, *A* and *K*, and then evaluating these derivatives at $E_o$

Then, $$\mathcal {F}=\left[ \begin{array}{ccc} k_2 & 0& 0 \\ 0& 0& 0\\ 0& 0& 0 \end{array}\right]$$

and $$\mathcal {V}=\left[ \begin{array}{ccc} \rho (1-k_3 )+(1-k_5 )s_1 +\mu & 0& 0 \\ -\rho (1-k_3)& c(1-k_7)+ (1-k_4)s+\mu _{A} & 0\\ 0& -(1-k_5)s_{1}-(1-k_4)s& (1-k_6)\delta +\mu _{K}\end{array}\right] .$$

After some calculation $$\mathcal {F}\mathcal {V}^{-1}=\left[ \begin{array}{ccc} \frac{k_2}{\rho (1-k_3 )+(1-k_5 ) s_{1}+\mu }& 0& 0 \\ 0& 0& 0\\ 0& 0& 0 \end{array}\right] .$$

Therefore the control reproduction number of the main system which represented in Eq. (1)

2 $$\begin{aligned} \rho (FV^{-1})=R_c=\frac{k_2}{\rho (1-k_3 )+(1-k_5 ) s_{1}+\mu }. \end{aligned}$$

From $R_c$ the increased effect of therapy(HAART) reduces prevalence of HIV/AIDS-related kidney diseases.

#### Local stability of the disease-free equilibrium

Local stability of the disease-free equilibrium point $E_o$ is determined by first finding the Jacobian matrix of the model system (1) with respect to each state variable (*S*, *H*, *A*, *K*). The populations have a unique disease-free steady state $E_o$ whenever the infectious disease is absent. To investigate the stability of HIV infection-free equilibrium, the Jacobian matrix is constructed

3 $$\begin{aligned} J(S,H,A,K)=\left[ \begin{array}{cccc} -\mu -\frac{k_2 H}{N} & -\frac{k_2 S}{N}& 0& 0 \\ 0 & -D_{1}+\frac{k_2 S}{N} -\mu & 0 & 0 \\ \frac{k_2 H}{N}& \rho (1-k_3 )& -(1-k_4)s+c (-1+k_7 )-\mu _{A}& 0 \\ 0& (1-k_5 ) {s_1}& (1-k_4) s & -(1-k_6) \delta -{{\mu }_k} \end{array}\right] \end{aligned}$$

where $$D_1=\rho (1-k_3 )+(1-k_5 )s_{1}$$

For disease free equilibrium Jacobian matrix of Eq. (3) represented as

$$J(E_{o})=\left[ \begin{array}{cccc} -\mu & -k_2& 0& 0 \\ 0 & -D_{1}+k_2 -\mu _{H} & 0 & 0 \\ 0& \rho (1-k_3 )& -(1-k_4)s+c (-1+k_7 )-\mu _{A}& 0 \\ 0& (1-k_5 ) {s_1}& (1-k_4) s & -(1-k_6) \delta -{{\mu }_k} \end{array}\right]$$

which gives the eigenvalues as follows;

$$\begin{aligned} \lambda _{1}= & -\mu \\ \lambda _{2}= & -((1-k_4)s+c (1-k_7 )+\mu _A) \\ \lambda _{3}= & -\rho (1-k_3 )+\frac{k_2 }{\mu }-(1-k_5)s_1-\mu _H \\= & R_{c}(\rho (1-k_3 )+(1-k_5)s_1+\mu _H)-(\rho (1-\alpha )+c (1-k_7 )+(1-k_5)s_1+\mu _H)\\= & (R_{c}-1)(\rho (1-k_3 )+(1-k_5)s_1+\mu _H)<0 {\textrm{if}} R_c<1\\ \lambda _{4}= & -(\mu _k+(1-k_6)\delta ). \end{aligned}$$

All the eigenvalues at HIV/AIDS infection-free are negative if $R_{c}<1$, therefore the dynamical system is locally asymptotically stable. It suggests that HIV can be eliminated from the community when $R_c<1$, which will reduce the cases of kidney disease, and when $R_c>1$, HIV will spread into the population, which will cause a rise in the number of cases of kidney disease.

#### Global stability of the disease-free equilibrium

To gain insights into the global stability of the disease free-equilibrium, the following results were presented

##### Theorem 3

*The disease free equilibrium point* $E_o$ *is global asymptotically stable if* $R_c<1$ *otherwise unstable*.

##### Proof

By using Lyapunov function such as $L:\mathbb {R}_{+}^{4}\longrightarrow \mathbb {R}_{+} \text {which defined as}$

$$\begin{aligned} L(S,H,A,K) & = a_{1} (S - S^{*} \ln S) + a_{2} (H - H^{*} \ln H) + a_{3} (A - A^{*} \ln A) + a_{4} (K - K^{*} \ln K) \\ & \Rightarrow \frac{{dL}}{{dt}} = a_{1} \left( {1 - \frac{{S^{*} }}{S}} \right)\frac{{dS}}{{dt}} + a_{2} \left( {1 - \frac{{H^{*} }}{H}} \right)\frac{{dH}}{{dt}} + a_{3} \left( {1 - \frac{{A^{*} }}{A}} \right)\frac{{dA}}{{dt}} + a_{4} \left( {1 - \frac{{K^{*} }}{K}} \right)\frac{{dK}}{{dt}} \\ & \Rightarrow \frac{{dL}}{{dt}} = [k_{2} - \mu - (1 - k_{3} )\rho - (1 - k_{5} )s_{1} ]H \\ & \Rightarrow \frac{{dL}}{{dt}} = [(\mu + (1 - k_{3} )\rho + (1 - k_{5} )s_{1} )](R_{c} - 1)H \\ \end{aligned}$$

$\frac{dL}{dt}\le 0$ if $R_c\le 1.$ Therefore, the disease-free equilibrium point is globally asymptotically stable whenever $R_c\le 1.$ $\square$

### Data

The onset of AIDS was first reported in Los Angeles in June 1981 and Tanzania’s Kagera region in 1983^39^. However in Tanzania, due to insufficient records, the documentation of cases did not commence until 1990. The HIV data for Tanzania in general population from 1990 to 2022 was acquired from the UNAIDS 2023 estimates. This dataset includes annual HIV estimates along with uncertainty bounds(low and high boundaries). The study considered a column detailing the estimated number of adults and children living with HIV in Tanzania. The data used in this study can be found in^40^.

## Numerical analysis

In this section, the model system (1) was solved numerically to validate the analytical results obtained in previous sections and to investigate the effect of Highly Active Antiretroviral Therapy (HAART) on HIV-related kidney disease dynamics. The mathematical model was analyzed in two scenarios: one where HAART has no impact on the infectious group and another where HAART affects the infectious group differently. Furthermore, we examined different compartment sizes and data sample sizes for parameter identifiability. This helps to determine the quality and nature of data needed to fit the model for the two cases considered.

### Parameter estimations with Markov chain Monte Carlo

The unknown parameters were obtained by fitting the model to the data using Markov Chain Monte Carlo (MCMC), as it allows for the simulation of model predictions while incorporating parameter uncertainty. The Adaptive Metropolis (AM) algorithm, implemented in the MATLAB MCMC toolbox^41^, was used for parameter estimation.

#### Implementation of MCMC toolbox in Matlab

To run MCMC sampling, two functions are required: the function that computes the solution of the model and the cost function. To compute the model solutions, the system of ODEs was solved numerically using the standard MATLAB’s ode45 solver which implements Fourth-Order Runge-Kutta (RK4) method. The cost function, which gives the sum of squared differences between the observations and the model outputs, is defined as:

$$S_{\text {sum}} = \sum _{i=1}^{N_r} (Y_i - \hat{Y}_i)^2,$$

where $N_r$ is the number of responses over which the sum of squares is computed, and $Y_i$ represents the observed data given as input to the MCMC model and $\hat{Y}$ represents the model-predicted response values.

The MCMC model requires initial guesses for the parameters. The initial guesses were generated by preliminary optimization using the fminsearch nonlinear optimizer. Additionally, bounds are specified for the parameters: probabilistic parameters are constrained between 0 and 1, while other rates are given positive bounds. The initial values and prior distributions specified for the estimated parameters are provided in Table 2. A proposal covariance matrix is also provided as part of the MCMC setup. Since the exact proposal covariance is unknown, reasonable initial proposal values are chosen to initiate the MCMC sampling. For this study, the adaptive MCMC algorithm^41^ was used. Adaptive MCMC enhances the traditional Metropolis algorithm by updating the proposal covariance during the MCMC run, using information from previously sampled points. In this case, we run the DRAM method for 50000 iterations, starting with a small proposal covariance 0.01$\textbf{I}$ and performing covariance adaptation at every 100th step. Recall that the MCMC approach involves generating candidate parameter values from a predefined proposal distribution. These candidate values are either accepted or rejected based on their likelihood, ensuring that the model outputs align closely with the observed data. Thus, outputs of the MCMC process include: Chains of parameter values sampled during each iteration. Cost function values that quantify the fit of the model to the data. To assess the quality of parameter estimation, the posterior distribution of the parameter chains is analyzed. A well-constrained parameter is indicated by a concentrated posterior distribution. Visualizing the chain mixing, such as plotting the chains and examining pairwise correlation plots, is a good practice to ensure that parameters are in agreement with the data and confined to a bounded region of the parameter space. Furthermore, the posterior distributions of the parameter chains are used to make predictions about the model’s responses. These predictions can be made within a $95\%$ credible interval by computing $2.5\%$ and $97.5\%$ quantiles of the posterior chain to reflect the uncertainties in the parameter estimates. This provides insights into the reliability of the model outputs under the given parameter uncertainties.

Using synthetic data to evaluate parameter identifiability (and uncertainties in parameter estimation) across different scenarios. The synthetic data was generated by introducing Gaussian noise to the model’s output using the function

$$y = f(x; \theta ) + \text {rand}(\text {size}(f(x; \theta ))) \cdot \sigma$$

where $f(x; \theta )$ is the solution of Eq. (1), $\theta$ represents the unknown parameters$(k_1-k_7)$, and $\sigma = 0.05$. As some parameter values are known from the literature, we focus on estimating those that are crucial not only for capturing local epidemiological conditions and healthcare accessibility but also for assessing the impact of HAART in the studied context. These include the recruitment rate ($k_1$), the effective transmission rate ($k_2$), and the overall efficacy of HAART ($k_3 - k_7$). In addition, focusing on parameters expected to vary significantly across populations helps address potential identifiability issues that could arise from estimating too many parameters simultaneously.

**Fig. 2:**
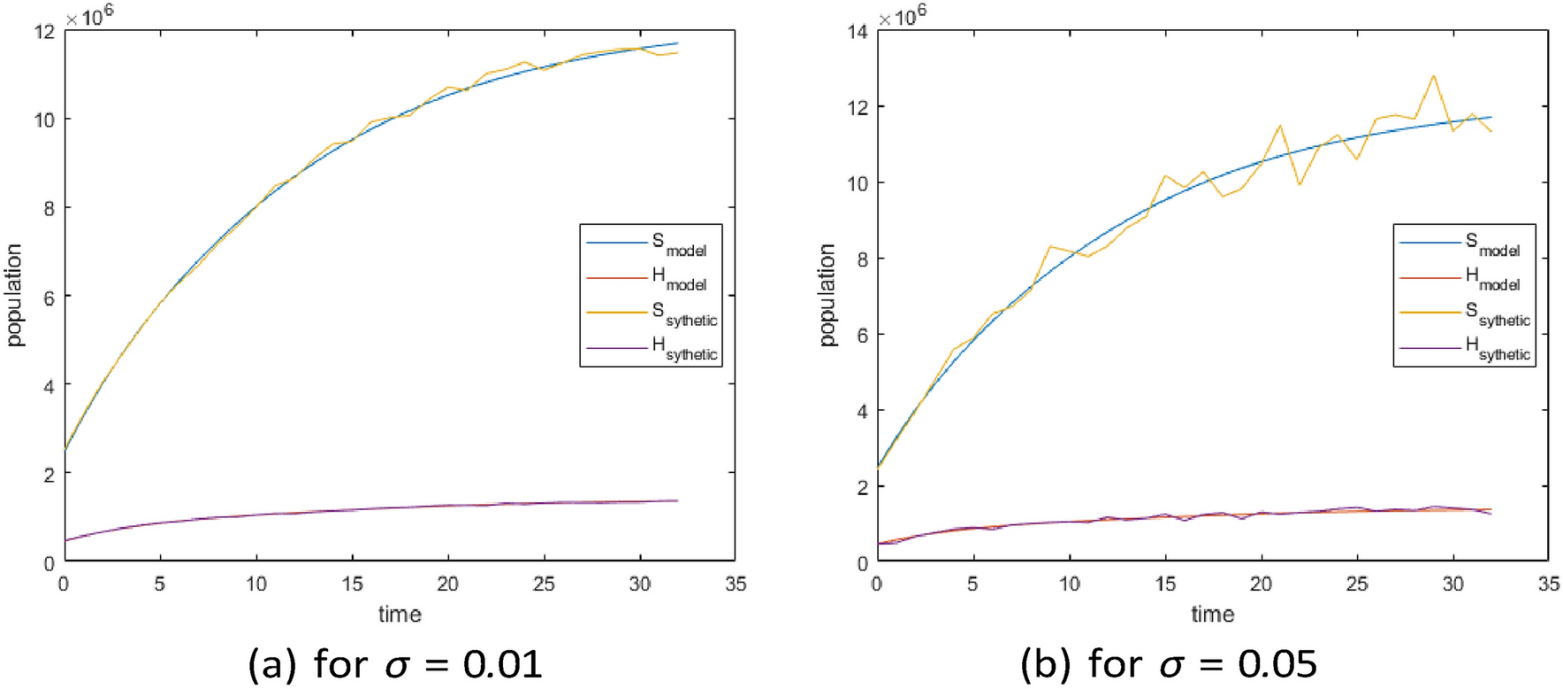
Synthetics data and model with different levels of noise by considering two compartments (Susceptible and HIV); (**a**) for $\sigma =0.01$ (**b**) for $\sigma =0.05$.

The value $\sigma =0.05$ was selected to match the observed data, ensuring that the generated data accurately reflects the characteristics of the real data (see Fig. 2). We ran 50, 000 MCMC simulations for each of the cases considered.

**Table 2:** Initial values and prior distributions for the estimated parameters.

Parameter	Initial value	Prior distribution
$k_1$	1410000	U$(0, \infty )$
$k_2$	1.15	U$(0,\infty )$
$k_3$	0.1	U(0, 1)
$k_4$	0.3	U(0, 1)
$k_5$	0.4	U(0, 1)
$k_6$	0.3	U(0, 1)
$k_7$	0.21	U(0, 1)

Most developing countries, such as Tanzania, implemented HAART late, and not all HIV-positive individuals have started using HAART. As a result, to ensure the model’s relevance, also considering that kidney problems in HIV-positive individuals are linked to both HIV infection and therapy usage, this study considers two scenarios when estimating the parameters. Case I assumes that no treatment or therapy affects any of the infection groups, while Case II assumes that therapy has varying impacts on each infection group.

#### Case I: When there is no HAART

In the model, all therapy effects are set to zero, and we estimate two parameters: the recruitment rate $k_1$ and the effective transmission rate $k_2$. This estimation is performed both when data is available for all compartments and when data is available for only two classes such as susceptible and HIV or one (HIV) compartment, as it is challenging to obtain comprehensive data from the general population in real life.

**Fig. 3:**
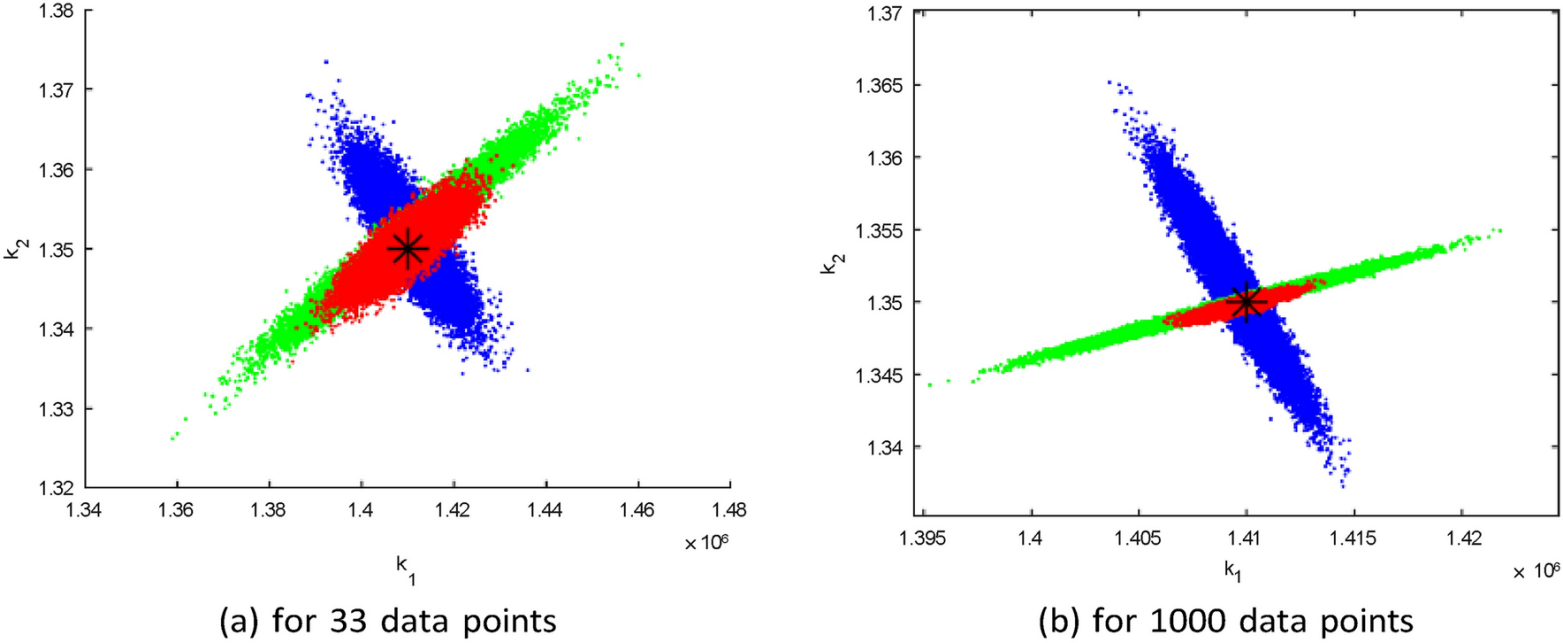
Pairwise chain parameters; red for four compartments, green for two compartments (Susceptible and HIV) and blue for one compartment (HIV).

**Fig. 4:**
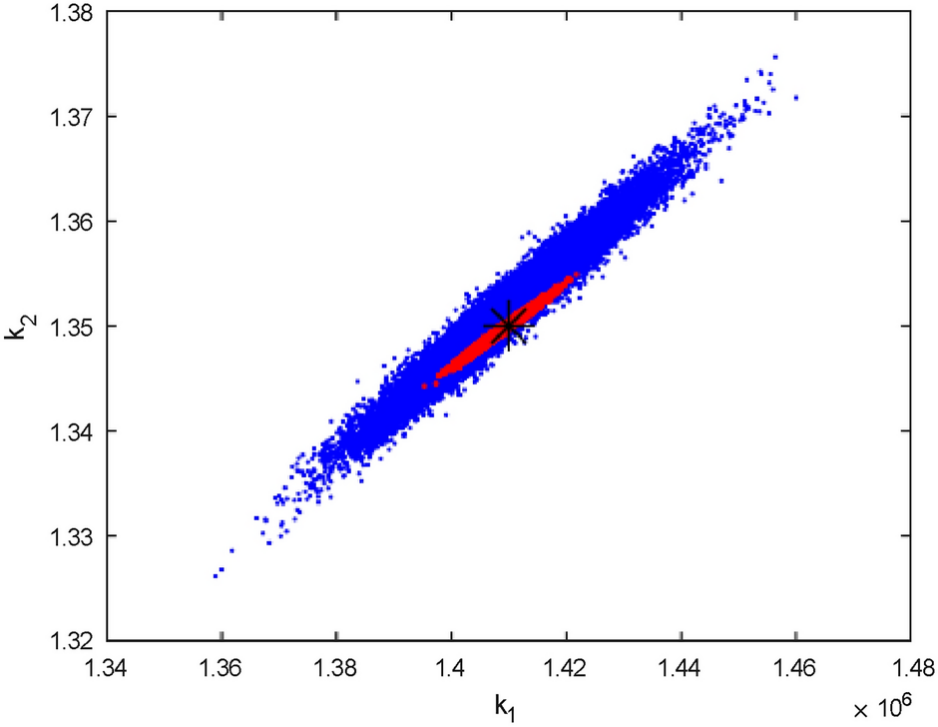
Pairwise chain parameters for two compartments (Susceptible and HIV) with different data set; red for 1000 data points and blue for 33 data points.

Figure 3 shows that parameters originating from a single compartment have a higher degree of uncertainty compared to parameters from four and two compartments. Additionally, a positive correlation is observed between these parameters when multiple compartments are employed in the analysis. Hence, expanding the dataset size can reduce parameter uncertainty. Furthermore, the number of sample points in the dataset impacts the degree of uncertainty in the parameters, as illustrated in Fig. 4. However, even with limited data points, it is possible to uniquely identify the recruitment rate and transmission rate in this model apparently because there are only two parameters to be identified in this case.

#### Case II: When HAART is implemented

The aim is to evaluate the effect of HAART on HIV/AIDS with related kidney disease populations and project the prevalence of the disease. The study examined the model system (1), taking into consideration the potential of having data for one, two, and four compartments, using both 33 and 1000 data samples since it would be challenging to gather data for all compartments in developing countries. In the model natural mortality rate, $\mu$ is fixed based on the life expectancy of Tanzanians. So according to database from World bank^34^, an average life expectancy is 67.3 years, hence $\mu =\frac{1}{67.3}$.

**Fig. 5:**
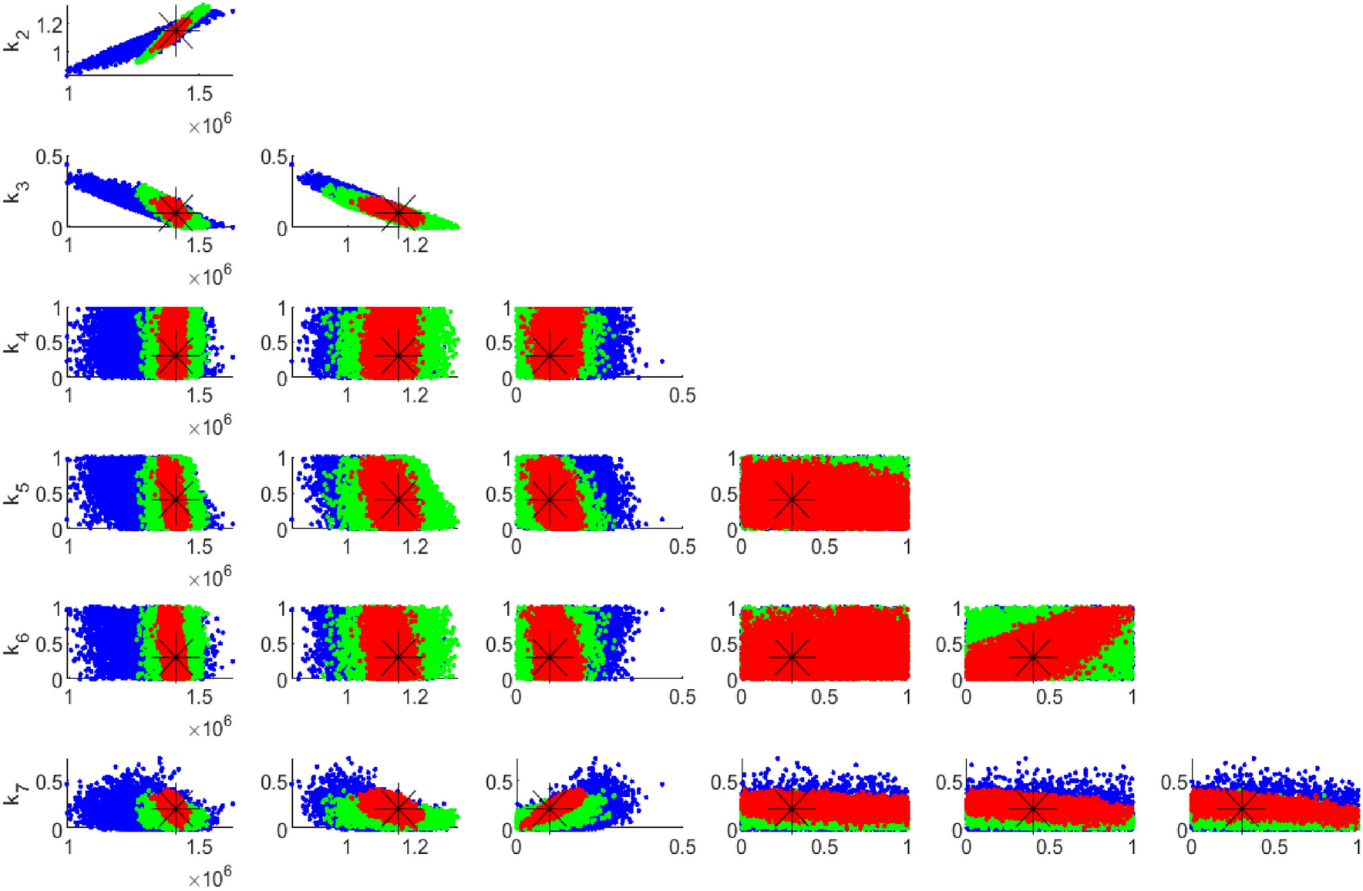
Pairwise chain parameters for 33 data sample; red for four compartments, green for two compartments, and blue for one compartment (HIV).

**Fig. 6:**
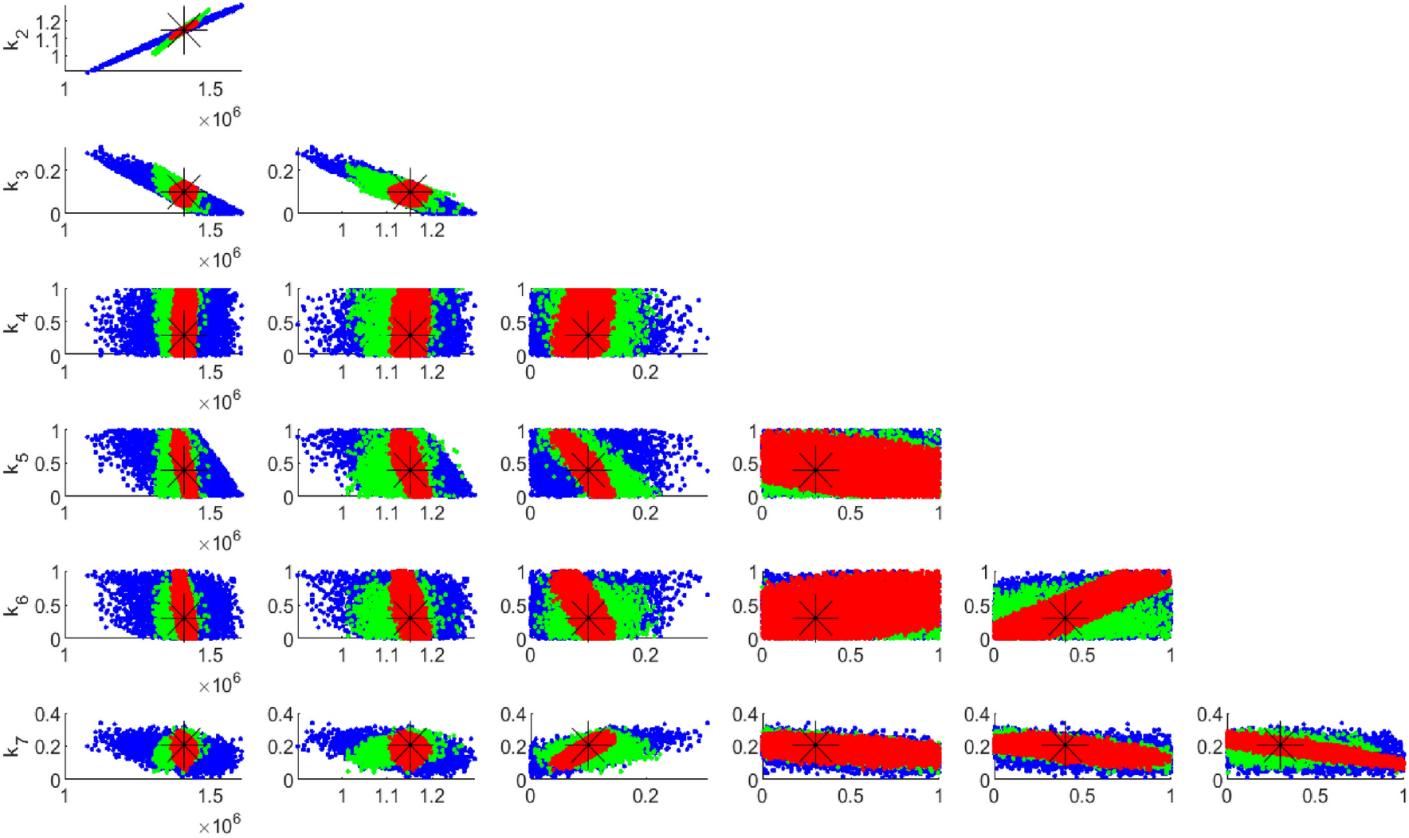
Pairwise chain parameters for 1000 data points; red for four compartments, green for two compartments, blue for one compartment (HIV).

Figure 5 compares the uncertainties in parameter estimation for different compartment sizes using 33 data points. It is evident that the smallest compartment size has the largest uncertainty in parameter identifiability. This observation is consistent with Fig. 6, which utilized 1000 data points. In both cases, whether the data points are 1000 or 33, the Figures demonstrate that having data for only one compartment is insufficient, as the majority of the parameters are not well identified.

**Fig. 7:**
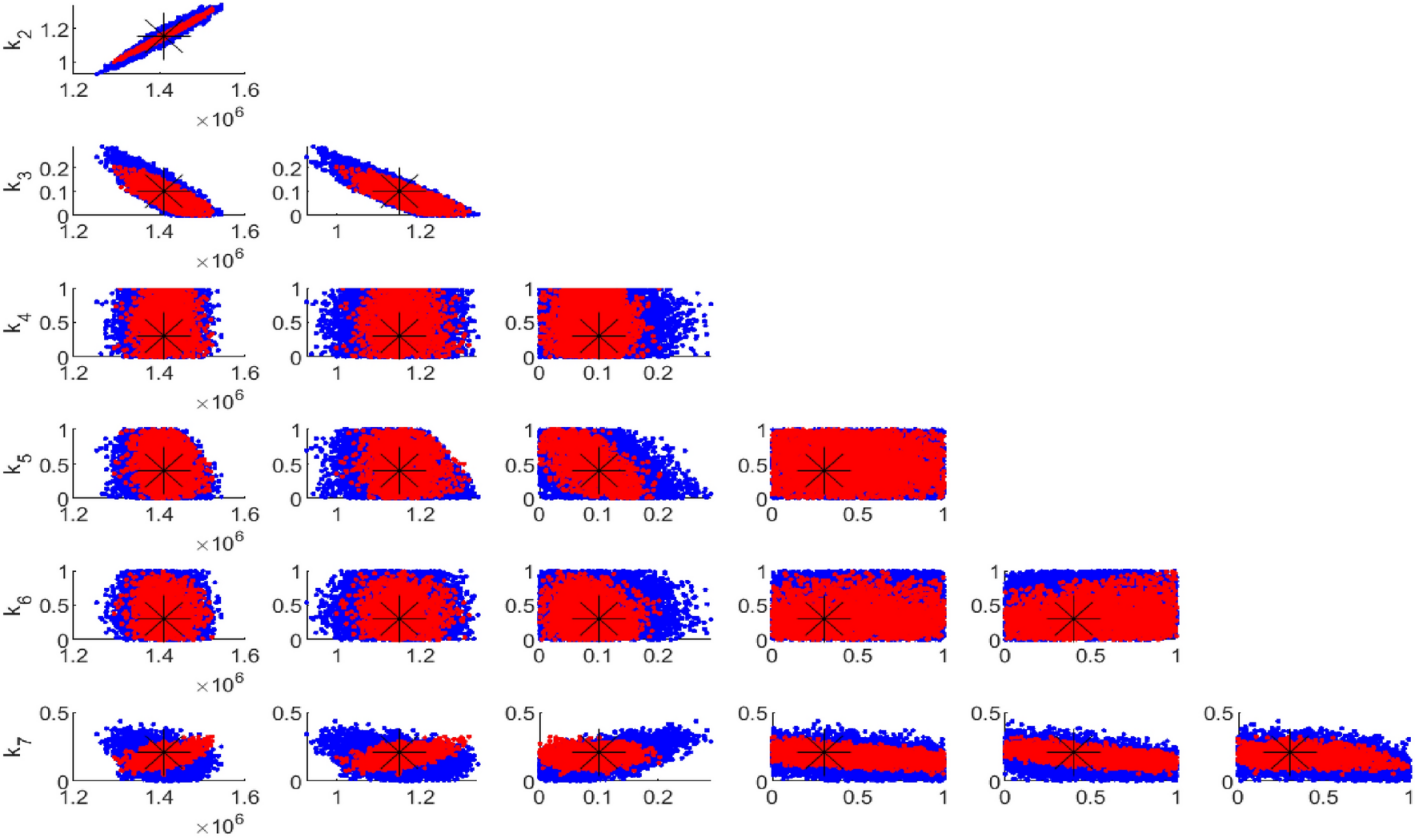
Pairwise chain parameters for two compartments with different data; red for 1000 data and blue for 33 data.

However, Fig. 7 highlights the difference in uncertainty levels between 1000 and 33 data points. Specifically, 33 data points are inadequate for identifying the seven unknown parameters of the model, even with data from two compartments, which are realistically easier to collect.

**Fig. 8:**
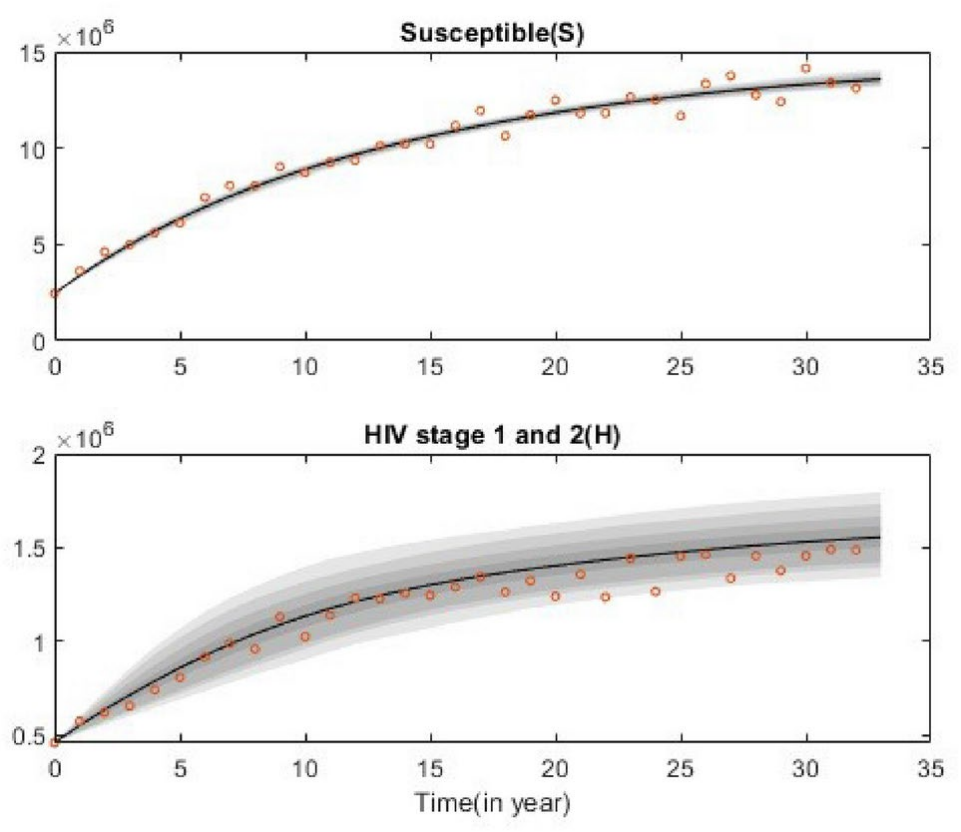
Plot of the synthetics data-fitted model solutions together with the uncertainties. Circles represent data points for susceptible and HIV in stages 1 and 2. Solid lines represent a posterior mean solution and grey areas represent solutions from different parameter uncertainties.

Figure 8 presents the predictive plot with synthetic data when 33 data points from two compartments are used. The predictive chain from different iterations is shown. In Fig. 8, the solid lines represent the median fits (the posterior means of the model parameters), while the lighter areas indicate the uncertainties in the prediction. Although not all parameters are uniquely identified, the predictions still follow the data trend, albeit with very large uncertainties.

Figures 5 and 6 reveal that incorporating more compartments in the analysis led to a reduction in parameter uncertainties for both sets of data samples.

The analysis with synthetic data suggests that when the treatment has no impact on infectious populations, the findings show that even with small data sets and sample sizes, parameter values are well identified. The results for seven parameters reveal that when the sample size was larger in terms of compartments and data points, the parameters were estimated to be better defined than when only one compartment was employed for analysis.

### Case study in Tanzania

In this section, the cumulative data of HIV cases in Tanzania since $1990-2022$ from UNAIDS was used to estimate the parameters of model (1).

#### Case I: When there is no therapy

In this case, all effects of HAART in the model (1) are set to zero and only two parameters remain for estimation. Based on the adaptive MCMC, the results of parameter estimation of the model (1) are given in Table 3.

**Table 3:** Fitted parameters with their $95\%$ confidence interval.

Parameter	Posterior mean	$95\%$ confidence interval
$k_1$	1707900	$[1697321-1793215]$
$k_2$	1.3234	$[1.2354-1.4326]$

**Fig. 9:**
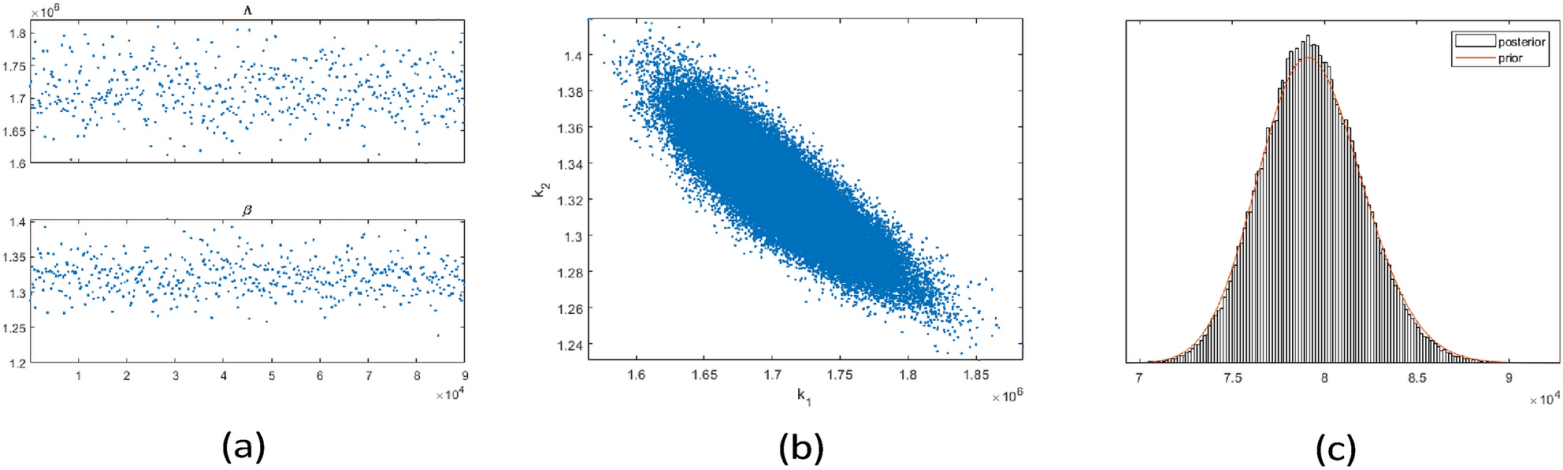
(**a**) Trace plot of mean value of the parameters. (**b**) Two-dimensional marginal posterior distributions of the model parameters. (**c**) Histogram of the chain for posterior error standard deviation with prior as a line.

Figure 9 shows that the parameters were well identified, similar to the synthetic case, and revealed a negative correlation between the recruitment rate of the susceptible population and the effective transmission rate.

**Fig. 10:**
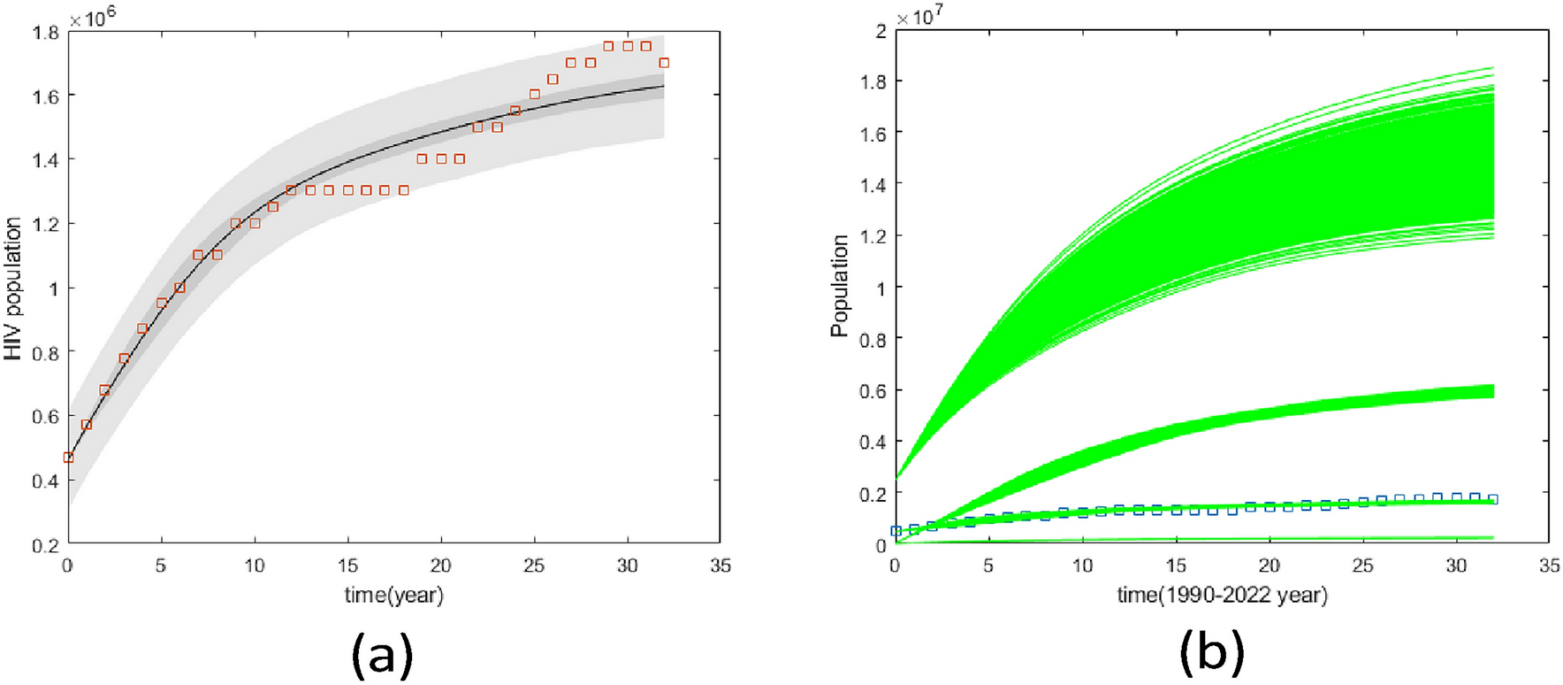
Fitted curve for HIV in stage one and two from 1990 to 2022. The squares denote the data and the solid lines show the median fits (the posterior means of the model parameters), while the lighter areas present the same uncertainty level in predicting new observations.

Figure 10a the predictive plot focuses on the HIV (H) compartment, showing the model’s estimated trajectory along with the $95\%$ posterior probability limits, which capture the uncertainty in model solutions. Figure 10b shows the predictive plots for all the other compartments derived from the posterior fits using data for the HIV(H) compartments. The predictive plot indicates a good fit with the data with reasonable uncertainty bounds.

#### Case II: When HAART is implemented

This study aims to evaluate the impact of HAART on HIV-related kidney diseases. This subsection is divided into two parts: the first part involves estimating parameters, such as the recruitment rate $(k_1)$, the effective transmission rate $(k_2)$, and the effects of HAART $(k_3-k_7)$ on infection groups by using the MCMC method. The second part uses these estimated parameters to project HIV-related kidney disease trends over a 60-year period (1990–2050), based on the UNAIDS report^42^, which warns of a potential rise in HIV cases by 2050 if prevention and treatment efforts are not strengthened. Additionally, this period aligns with Tanzania’s Vision 2050, which includes decentralizing healthcare services to district levels to reduce the burden on regional and referral hospitals^43^.

HIV data from Tanzania$(1990-2022)$ were used to assess the effect of HAART on individuals with HIV/AIDS-related kidney diseases. Due to the four-compartment structure of the model (1), only a single dataset of HIV-infected individuals from 1990 to 2022 was available. Therefore, in MCMC, the model solution was set to provide a single solution for HIV compartments, which is compared with the data to estimate the parameters.

**Fig. 11:**
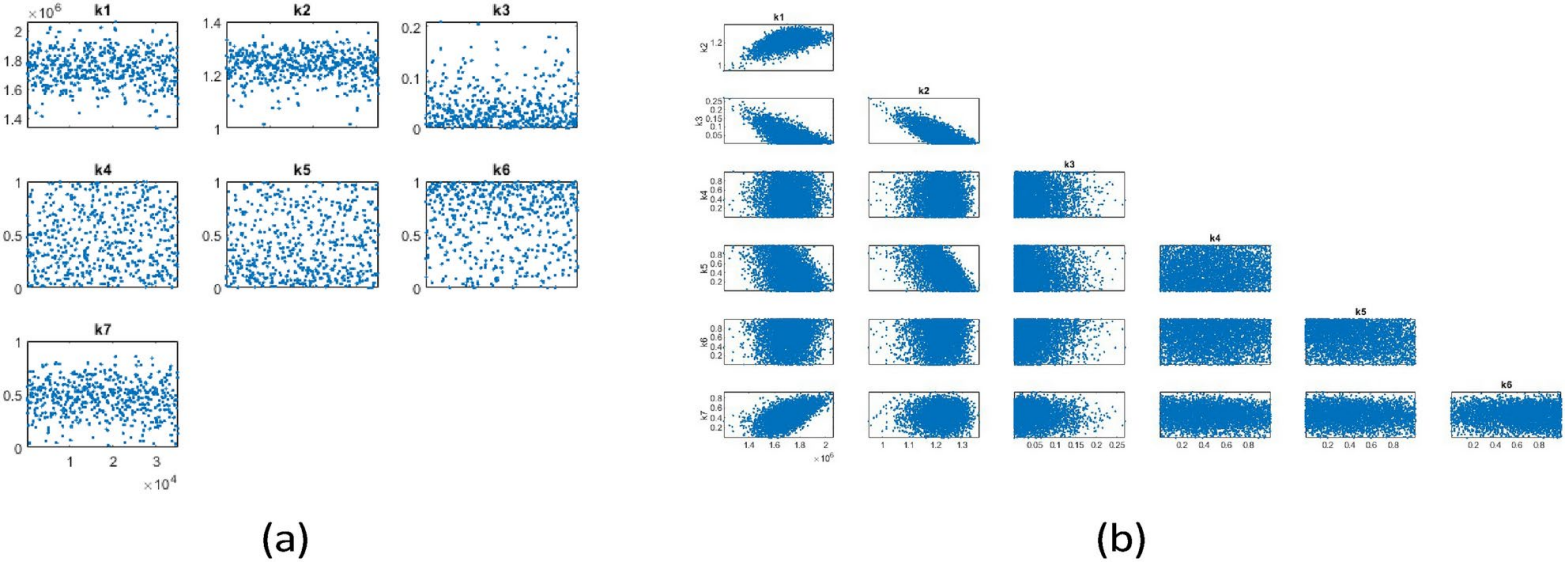
Results of parameter estimation for Case II parameters: (**a**) Trace plot (**b**) Pairwise correlation.

Figure 11a illustrates one-dimensional parameter chains, with most of the parameters being well identified. This convergence is achieved by applying a positivity constraint to parameters $k_3$ to $k_7$, which describe the effects of HAART and range from 0 to 1. Although not all parameters were uniquely identified, meaningful correlations occurred between certain parameters as shown in Fig. 11b. For instance: a negative correlation was found between $(k_2, k_3)$, suggesting that effective HAART reduces viral loads, which in turn lowers the chances of transmitting the virus to others. Similarly, a negative correlation between $(k_2, k_5)$ suggests that effective HAART reduces viral load, thereby lowering the transmission rate and simultaneously preventing the progression to kidney disease. For $(k_1, k_3)$, the negative correlation suggests that effective HAART can contribute to broader public health efforts that reduce the exposure and infection rates among susceptible individuals, thus potentially leading to a meaningful negative correlation indirectly.

A positive relationship exists between $(k_1, k_2)$. This positive relationship between the recruitment rate of susceptible individuals and the effective transmission rate of HIV is meaningful and can be expected under several scenarios: one notable scenario is that as more individuals become susceptible, the potential for HIV transmission increases, leading to a higher effective transmission rate. Additionally, a positive relationship was seen between $(k_1, k_7)$. This positive relationship between the recruitment rate of susceptible individuals and the effect of HAART in reducing mortality due to AIDS may indicate that effective HAART programs, while reducing AIDS mortality, are accompanied by increased awareness, testing, and identification of at-risk individuals, leading to a higher recorded rate of susceptibility. The results for parameter estimations are represented in Table 4.

**Table 4:** Fitted parameters with their $95\%$ confidence interval.

Parameters	Posterior mean	$95\%$ confidence interval
$k_1$	1742500	$[1553637-1933530]$
$k_2$	1.2327	$[1.1082- 1.3171]$
$k_3$	0.0385	$[0.0012-0.1355]$
$k_4$	0.4557	$[0.0154- 0.9660]$
$k_5$	0.4133	$[0.0011- 0.9532]$
$k_6$	0.6375	$[0.0491- 0.9889]$
$k_7$	0.4597	$[0.1114- 0.7547]$

The posterior mean and $95\%$ confidence interval range of the parameters in Table 4 were determined based on the posterior mean and the $2.5\%$ and $97.5\%$ percentiles of the chain parameters obtained from the mcmcrun function after running 34000 simulations.

**Fig. 12:**
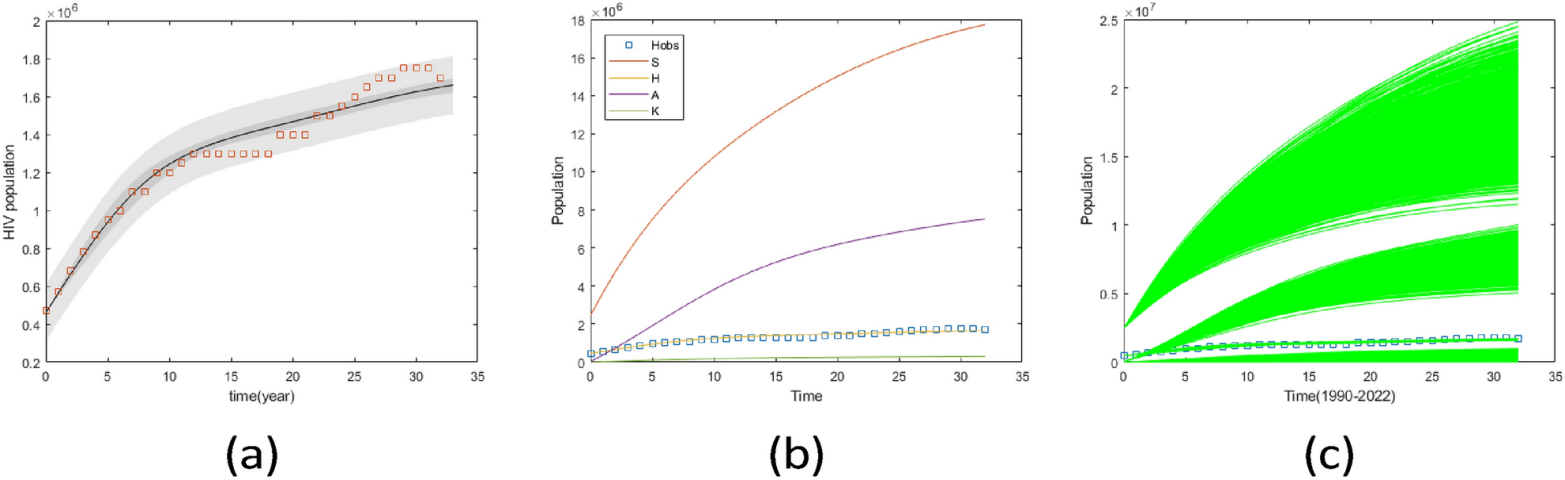
Predictive envelopes of the model with seven parameters from $1990-2022$. (**a**) represents fitted HIV data with a posterior range of $95\%$ confidence interval, (**b**) represents a population with different compartments by using posterior mean, and (**c**) predictive graph with random posterior value for susceptible, HIV, AIDS, and HIV/AIDS-related kidney diseases.

**Fig. 13:**
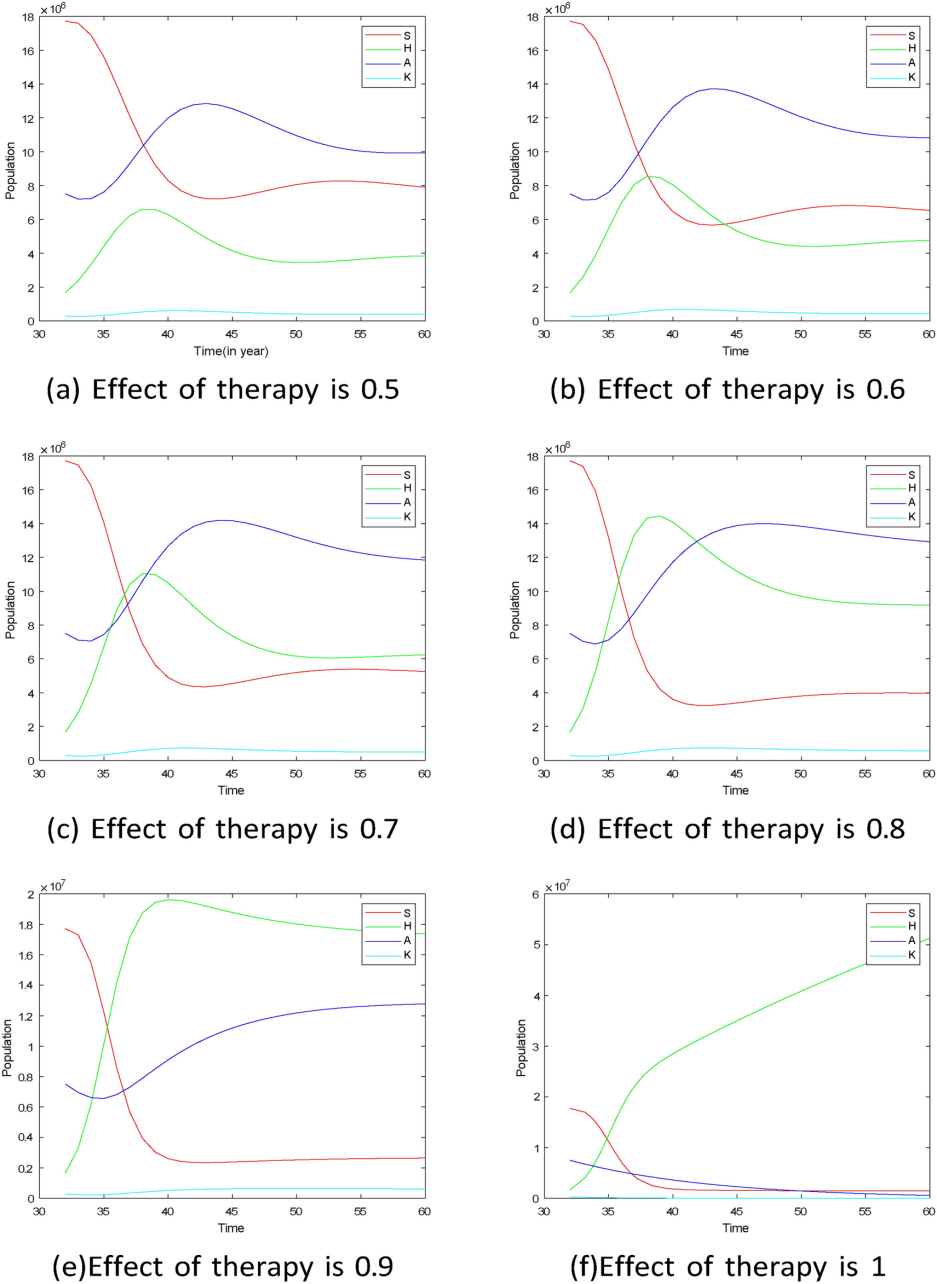
Projection of the total population considering various therapy effects, also including a scenario where the therapy is completely effective (effect of therapy is 1), from 2023 to 2050.

Additionally, the predictive plot presented in Fig. 12 indicates a good fit with the data with reasonable uncertainty bounds even when not all parameters were well identified. Based on the parameter values provided in Tables 1 and 4, the estimated control reproduction number is 1.5446, with a $95\%$ confidence interval ranging from 1.3468 to 1.8774. The result shows $R_c>1$ means that, on average, each HIV-infected individual is transmitting the virus to more than one person and the disease could become endemic within the population.

**Fig. 14:**
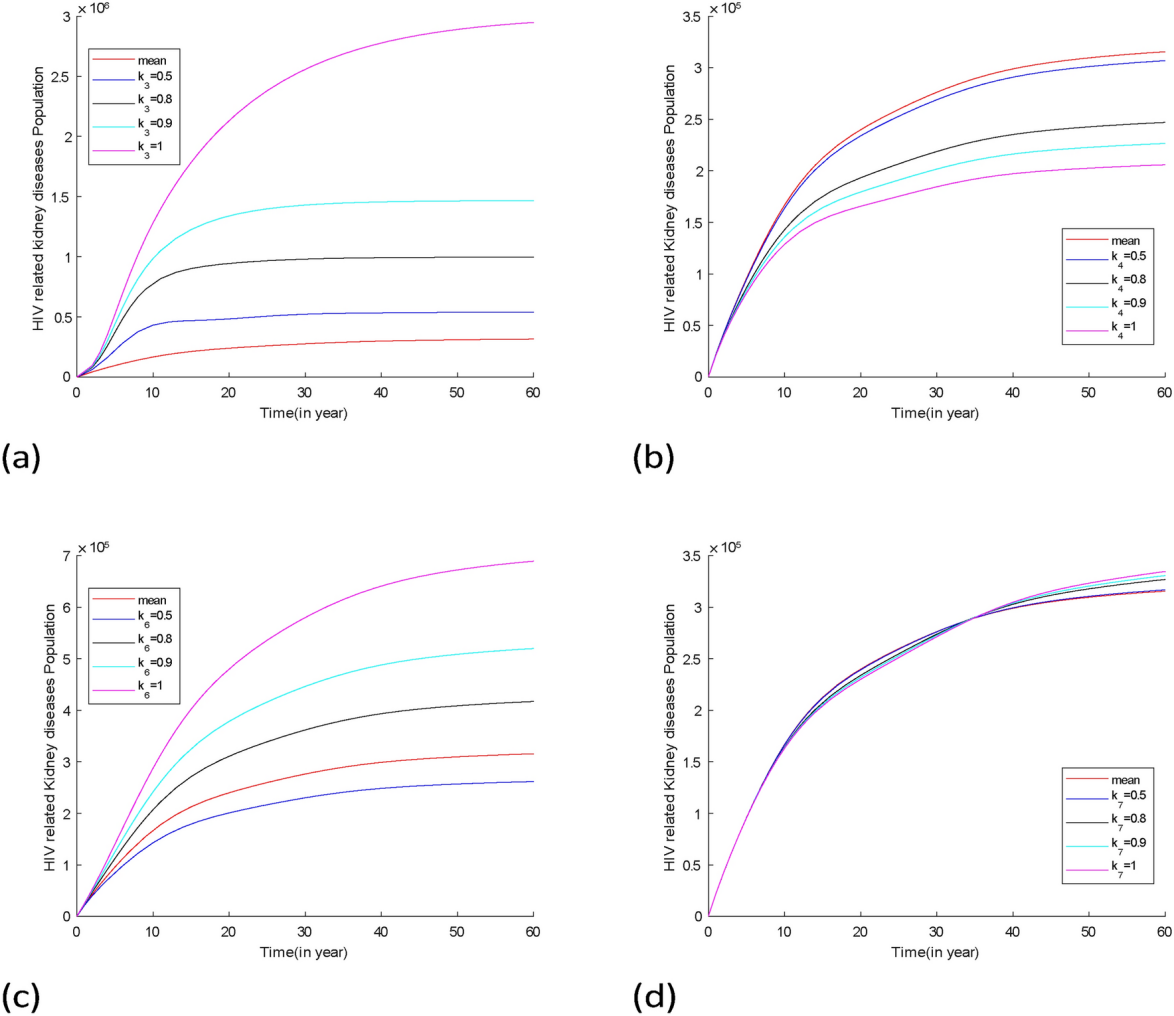
Projection of HIV-related kidney diseases with different effects of therapy based on the confidence interval and posterior mean in Table 4. (**a**) effect of HAART to block the progression from HIV to AIDS $(k_3)$ varies, (**b**) effect of HAART to reduce the progression from AIDS to HIV to HIV+ kidney diseases varies $(k_4),$ (**c**) effect of HAART to reduce mortality due to HIV+ kidney diseases varies $(k_6),$ (**d**) effect of HAART to reduce mortality due to AIDS $(k_7)$ varies. For all cases, the posterior means of the non-varied parameters were used as fixed values for the simulations.

**Fig. 15:**
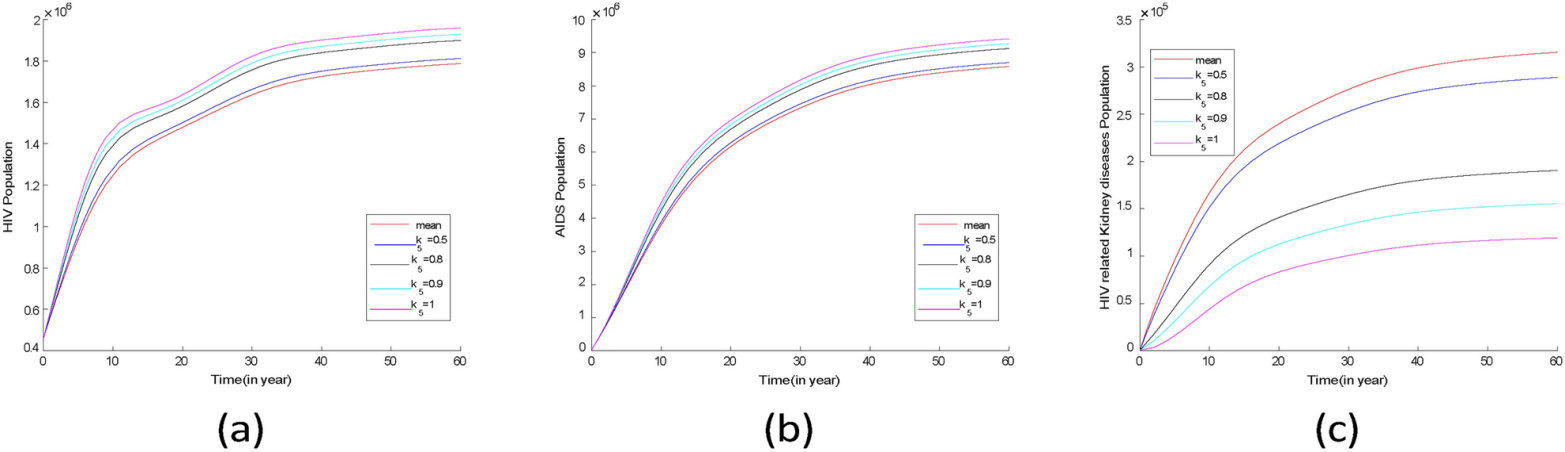
Plots of projection of infectious groups, when the effect of therapy in reducing progression from HIV to HIV-related kidney diseases ($k_5$) varies within the confidence interval values provided in Table 4, other parameters are assigned their posterior mean values. Additionally, the analysis extends to consider the scenario where therapy is fully effective ($k_5=1$).

The study investigates the prevalence of HIV-related kidney disease, considering different levels of HAART effectiveness while keeping other parameters constant, to understand the dynamics of the disease in the population. Simulation from Figs. 13, 14 and 15 show the effect of variations of HAART effect to the infected population. Figure 13 shows that after twenty years, the population of all infected groups will reach equilibrium when HAART has a $50\%$ efficiency. However, when HAART efficacy is between $50\%$ and $80\%$, the frequency of HIV+ESRD would grow dramatically for the first three years before declining to an endemic level. The model shows that strong gains can be achieved when HAART can simultaneously reduce entry into the AIDS population as well as reduce progression to HIV+ESRD from infected groups, even if its effectiveness is lower$(50\%)$. Also, Fig. 13f shows that when the efficacy of therapy is perfect the population of people living with HIV will increase but the population of other infectious groups will decrease.

Figure 14 shows the projection of HIV-related kidney diseases dynamic with different effects of therapy when other parameters are kept constant. The result shows that the dynamics of the diseases will increase when the effect of HAART to block the progression from HIV to AIDS and the effect of HAART to reduce mortality due to HIV-related kidney disease range from $0.6-0.9$ as shown in Fig. 14a,c. However, when the effect of HAART to reduce mortality due to HIV-related kidney disease is 0.5 the prevalence of diseases decreases before reaching the equilibrium. Also, it shows that when the effect of HAART to block progression from AIDS to HIV-related kidney diseases is between 0.5 and 1, the prevalence of diseases will decrease and reach equilibrium after a few years. The dynamics of diseases will be expected to decrease and increase before reaching equilibrium as shown in Fig. 14d when the effect of HAART on reduced mortality due to AIDS varies.

Figure 15 shows the projection of infectious groups when there is a variation of the effect of HAART to reduce the progression from HIV to HIV-related kidney diseases. It shows that the HIV and AIDS population will increase while the population of individuals with HIV-related kidney diseases decreases before reaching equilibrium. However, the infected population will not decrease to zero. This shows that to reduce the diseases in the community other control measures are required to be considered.

#### Comparison of two cases in infectious group

This study evaluates the impact of HAART on HIV-related kidney diseases in the general population. This subsection compares two scenarios: one without the introduction of HAART and the other with HAART implemented, to evaluate its effects on the general population. Furthermore, the study includes a projection to analyze the dynamics of the infection groups for both scenarios.

**Fig. 16:**
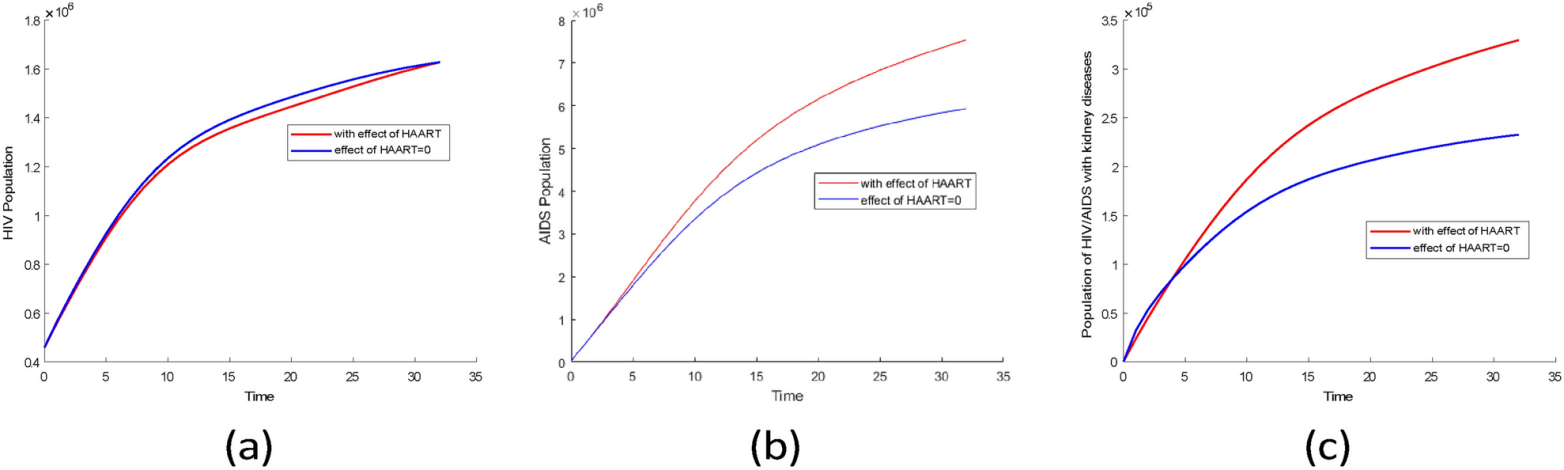
Comparison plots of infection groups over 33 years for two scenarios using the posterior mean values from Tables 3 and 4. (**a**) HIV without symptom population, (**b**) AIDS population, (**c**) population of HIV/AIDS with kidney diseases.

**Fig. 17:**
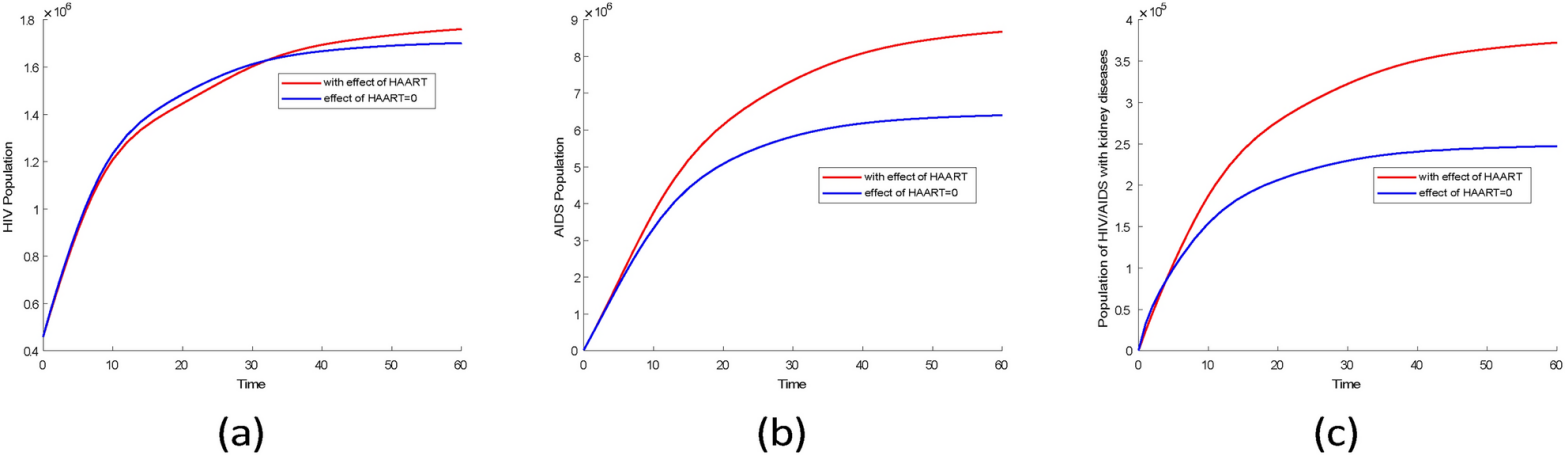
Plots of projection of comparison of infectious groups for two scenarios using the posterior mean values from Tables 3 and 4. (**a**) HIV without symptom population, (**b**) AIDS population, (**c**) population of HIV/AIDS with kidney diseases.

The mean values from Tables 3 and 4 were used to simulate two scenarios for comparison. The results indicate that there was little difference in the prevalence of the HIV population during the first 33 years, as shown in Fig. 16a. However, after 33 years, the prevalence of the HIV population increased significantly in the scenario where HAART was effective as represented in Fig. 17.

From Fig. 16b, in the scenario where there is HAART, the AIDS population is higher compared to scenarios where there is no HAART. This indicates that HAART has improved the lives of people with AIDS by reducing mortality in this group. Similar findings were reported in^44^ research, which showed a decline in AIDS-related mortality after HAART was introduced.

According to Fig. 16c the first four years, the prevalence of HIV-related kidney diseases is lower when there is the effect of HAART compared to when there is no effect. However, over time, the prevalence of HIV-related kidney diseases started to rise significantly when there is HAART. This indicates that kidney problems in HIV patients are not only caused by the virus itself but also by certain HAART medications. Research conducted by^45^ supports this finding, showing that prolonged HAART use can lead to decreased kidney function. Additionally^46^, suggests that extended use of HAART is linked to an increased risk of chronic kidney diseases.

For both cases, the infectious groups reached an equilibrium state as shown in Fig. 17. This result shows an increase of infectious groups and also kidney diseases among HIV/AIDS individuals. Therefore, to reduce the prevalence of kidney disease among HIV/AIDS group measures should be taken to reduce HIV infection and also to develop HAART which will reduce the effect on kidneys.

**Fig. 18:**
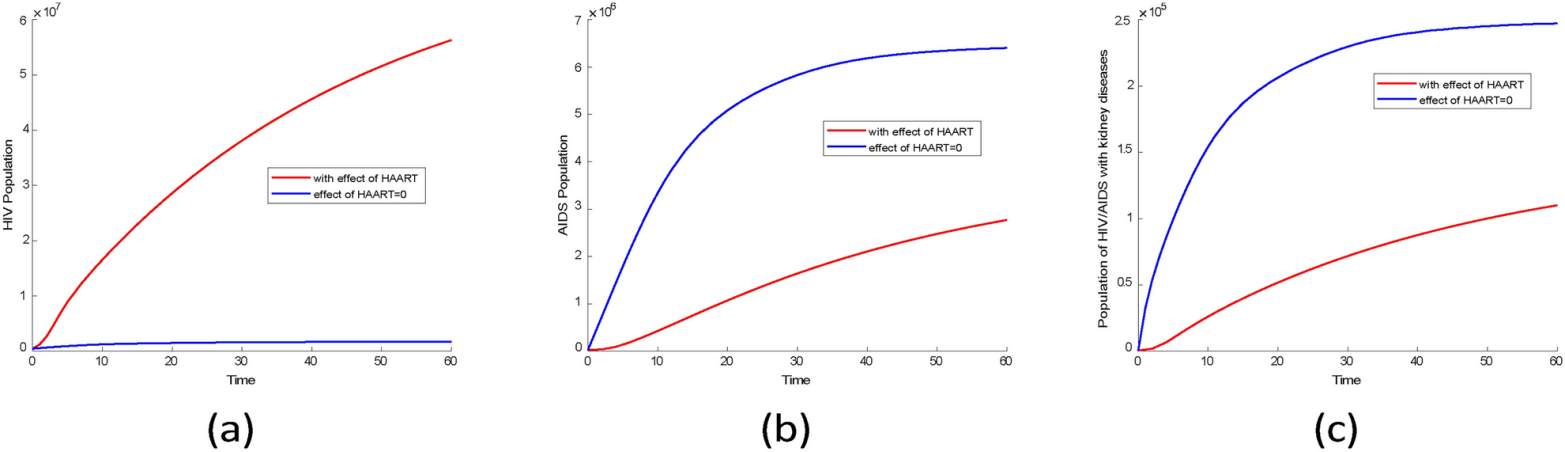
Comparison plots of infection groups for two scenarios: one with no HAART and the other where the effect of HAART is greater than 0.9. (**a**) HIV without symptom population, (**b**) AIDS population, (**c**) HIV/AIDS related kidney diseases population.

Figure 18 shows that when HAART is more than $90\%$ effective, the HIV population increases, but without HAART, the HIV population is smaller. This is different from AIDS and HIV-related kidney disease groups, which have higher populations without HAART. This indicates that HAART can reduce AIDS and HIV-related kidney disease cases but cannot eliminate these diseases, as shown in Fig. 18b and c.

### Sensitivity analysis

Sensitivity analysis is used to assess how changes in the model input parameters affect the model output. In this subsection, a sensitivity analysis was conducted to explore how uncertainties in parameter estimation influence the control reproduction number $(R_c)$ generated in Eq. (2). This includes assessing the impact of parameters such as the effectiveness of HAART in slowing the progression from HIV to HIV-related kidney disease $(k_5)$ and other parameters affecting $R_c$. Sensitivity analysis determines the influence of each parameter on the $R_c$ while keeping all other parameters fixed. A positive sensitivity index implies that an increase in the parameter leads to an increase in the control reproduction number, while a negative index indicates that an increase in the parameter results in a decrease of the control reproduction number.

Sensitivity index of variable $R_c$ that depends on parameter *p* is defined as $SI(p)=\frac{\partial R_c}{\partial p}*\frac{p}{R_c}$ as described by^47^. For example, the sensitivity index of $k_2$ is given by $SI(k_2)=\frac{\partial R_c}{\partial k_2}*\frac{k_2}{R_c}=+1$, also $SI(\rho )=\dfrac{-k_2(1-k_3)}{\rho (1-k_3)+(1-k_5)s_1+\mu }$ . Other indices are computed using the same approach Table 4.

We calculated the sensitivity index for each parameter based on the control reproduction number using parameter distributions obtained from MCMC sampling. Specifically, for each set of parameters sampled, the sensitivity index was calculated and the resulting distribution of indices was used to determine the mean and 95% confidence intervals. This approach quantifies the uncertainties in the sensitivity indices, reflecting the variability in parameter estimates. Table 5 shows the mean sensitivity indices of $R_c$ for the parameters along with their confidence intervals 95%.

**Table 5:** Sensitivity indices of $R_c$ with respect to parameters in range.

Parameters	Mean sensitivity index	Range in $95\%$ confidence interval
*k*2	$+1$	[1, 1]
$\rho$	$-0.9177$	$[-0.9748, -0.8765]$
*k*3	0.0381	[0.0012, 0.1423]
$s_1$	$-0.0636$	$[-0.1054, -0.0055]$
*k*5	0.0466	[0.0011, 0.1128]
$\mu$	$-0.0187$	$[-0.0209, -0.0174]$

The negative sign in sensitivity indices shows that the parameters are inversely proportional to $R_c$. This means that a decrease(increase) of any parameter $\rho , \mu$ and $s_1$, while other parameters are constant, will cause an increase(decrease) in $R_c$ and thereby lead to an increase (decrease) in the spread of disease in the population. On the other hand, parameters $k_2,k_3,k_5$ have a positive sensitivity index and hence increase (decrease) while other parameters are fixed will cause an increase(decrease) in $R_c$.

**Fig. 19:**
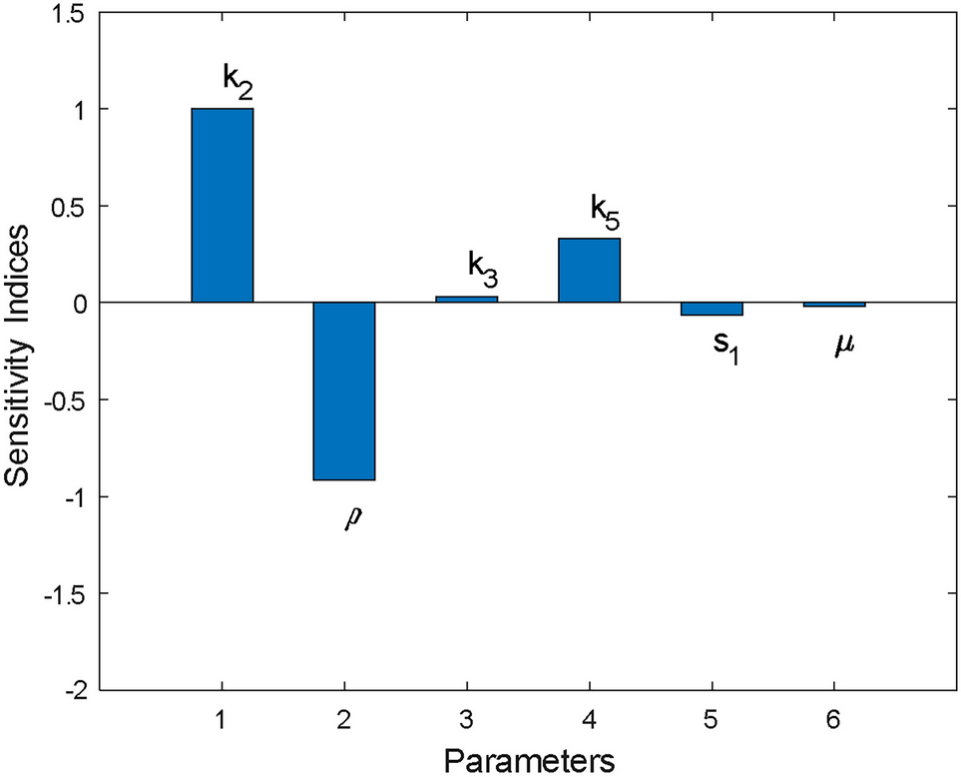
Sensitivity indices by using the posterior mean value from Table 5.

The result in Fig. 19 shows that the force of infection $(k_2)$, progression rate from HIV to AIDS $(\rho )$ and, the effect of HAART to block the progression from HIV to HIV-related kidney disease $(k_5)$ have the greatest impact on the dynamic of HIV-related kidney diseases. Also, the control reproduction number $(R_c$ will decrease with an increase of the effect of HAART ($k_3$ and $k_5$). Therefore, to reduce the prevalence of HIV-related kidney diseases, this study recommends that increasing the effect of therapy that will reduce the progression rate to the AIDS group and progression from HIV to HIV-related kidney diseases will reduce the prevalence of HIV-related kidney disease in the community.

## Discussion

In this study, a mathematical model of nonlinear differential equations on HIV/AIDS-related kidney diseases that incorporates treatment (HAART) at each stage of HIV infection was developed and examined. The positivity and boundedness are demonstrated to have epidemiological significance. In order to identify the risk factors for disease in a general population, the reproduction number is $(R_c)$ computed. Based on the Tanzania HIV data from 1990 to 2022, the calculated $(R_c)$ value 1.5446 $\left( {(R_{c} ) > 1} \right),$ indicating that each infected individual, on average, transmits the virus to more than one person. This finding underscores the urgency of public health interventions to curb the spread of the virus. A sensitivity analysis of the parameters was carried out to investigate the impact of each parameter on the infection groups, based on $(R_c)$. The effect of HAART in preventing the progression from HIV to HIV-related kidney disease was found to be more significant than its impact on slowing the progression from HIV to the AIDS stage. Additionally, the effective transmission rate was identified as the most sensitive parameter. These findings underscore the critical role of HAART in reducing or eliminating the risk of kidney disease among HIV-positive individuals. Therefore, prioritizing parameters with the highest sensitivity indices can serve as an effective strategy for managing the epidemic within the population.

MCMC method was used for parameter estimation. The study analyzes synthetic and real data on HIV prevalence in Tanzania from 1990 to 2022 to assess the model parameters under different scenarios. Using synthetic data was important for testing parameter identifiability because it allowed us to check how well the model could estimate parameters under various data availability conditions. The analysis showed that when therapy was not included, even small datasets provided reliable estimates. However, for the more complex scenario involving therapy, larger datasets and data covering more compartments were necessary to accurately estimate all seven parameters.

The numerical analysis of HIV data from Tanzania’s general population showed that HAART effectively reduces the progression from HIV to the AIDS stage, as well as to kidney diseases.The study of^48^ found that effective control of HIV through HAART use, moderate condom compliance, and reduction in sexual contact are essential for reducing transmission in men who have sex with men (MSM) population. Also, the rate of progress from HIV to HIV-related kidney disease is higher compared to the rate of progress from AIDS to HIV-related kidney disease. This result is similar to other articles^13,15,49^ which found that renal disease among HIV individuals does not depend on CD4 count. Therefore, as the population grows, there will be less disease-related mortality as well. The population of individuals with HIV/AIDS-related kidney diseases will be reduced to zero once HAART is completely effective, and this will lessen the burden on society.

The projection results is based on the current treatment rates and the effectiveness of HAART suggests that HAART treatment should be able to prevent the progression of HIV/AIDS-related kidney diseases and reduce disease-related deaths, by decreasing the number of individuals with kidney diseases associated with HIV/AIDS. Furthermore, the findings indicate that a 90% improvement in HAART effectiveness in preventing the progression from HIV to HIV-related kidney diseases could significantly lower the prevalence of HIV-related kidney diseases. Achieving a 90% improvement in HAART effectiveness in Tanzania is an ambitious goal, yet it comes with challenges. Factors such as patient adherence, drug resistance, access to advanced HAART treatments, and consistent monitoring of kidney function are all crucial. To make this goal achievable, it is necessary to strengthen healthcare systems, enhance education on adherence, and ensure a consistent supply of quality HAART. These findings have implications for the public, therefore the study recommended that policymakers and clinicians focus on improving HAART, which reduces the progression of kidney disease associated with HIV/AIDS, HAART access, and increasing monitoring for kidney diseases among HIV patients in Tanzania.

Future studies may expand the model to include time-varying parameters, including other strategies and measures that lower the chance of developing kidney disease to HIV/AIDS individuals, cost-effectiveness analysis, and also take into account fractional and stochastic differential models.

## Data Availability

Data will be made available upon reasonable request. Miracle Amadi (miracle.amadi@lut.fi) is the corresponding author and should be contacted if data is needed.

## References

[1] Huo, H.-F. & Chen, R. Stability of an HIV/AIDS treatment model with different stages. *Discret. Dyn. Nat. Soc.* **2015**, 630503 (2015).

[2] Teklu, S. W. & Rao, K. P. HIV/AIDS-pneumonia codynamics model analysis with vaccination and treatment. *Comput. Math. Methods Med.* **2022**, 3105734 (2022). 35069778 10.1155/2022/3105734 PMC8767370

[3] UNAIDS, D. Report. Retrieved February (2021).

[4] Lee, S. H. et al. Trends of mortality and cause of death among HIV-infected patients in Korea, 1990–2011. *J. Korean Med. Sci.* **28**(1), 67–73 (2013). 23341714 10.3346/jkms.2013.28.1.67 PMC3546107

[5] Alfano, G. et al. Kidney disease in HIV infection. *J. Clin. Med.* **8**(8), 1254 (2019). 31430930 10.3390/jcm8081254 PMC6722524

[6] CDC: Chronic kidney disease in the united states, 2019. Atlanta, GA: US Department of Health and Human Services, Centers for Disease Control and Prevention **3** (2019).

[7] Abraham, A. G. et al. End-stage renal disease among HIV-infected adults in north America. *Clin. Infect. Dis.* **60**(6), 941–949 (2015). 25409471 10.1093/cid/ciu919 PMC4357817

[8] Gudaz, H., Ogu, H. A. & Schwartz, E. J. Long-term dynamics of the kidney disease epidemic among HIV-infected individuals. *Spora J. Biomath.* **6**(1), 52–60 (2020).

[9] Samje, M., Youego, E., Kefeyin, T. & Lukong, H. Effects of highly active antiretroviral treatment on liver and renal functions of HIV-infected patients attending the day care clinic of the Bamenda regional hospital, Cameroon. *Afr. J. Clin. Exp. Microbiol.* **21**(4), 318–327 (2020).

[10] Jager, K. J. & Fraser, S. D. The ascending rank of chronic kidney disease in the global burden of disease study. *Nephrol. Dial. Transplant.* **32**((suppl–2)), 121–128 (2017). 10.1093/ndt/gfw330 28201666

[11] Mapesi, H., Kalinjuma, A. V., Ngerecha, A., Franzeck, F., Hatz, C., Tanner, M., Mayr, M., Furrer, H., Battegay, M., Letang, E., *et al.* Prevalence and evolution of renal impairment in people living with HIV in rural Tanzania. In: Open forum infectious diseases, vol. 5, p. 072 (Oxford University Press, 2018). 10.1093/ofid/ofy072 PMC5912087 29707599

[12] Kilonzo, S. B., Seiffudin, A. T., Bakshi, F. A. & Gunda, D. W. Renal dysfunction among adult patients in Mwanza, Tanzania: Prevalence, outcomes and associated factors. Tanzania J. Health Res. **18**(3) (2016).

[13] Mwemezi, O., Ruggajo, P., Mngumi, J. & Furia, F. F. Renal dysfunction among HIV-infected patients on antiretroviral therapy in Dar es Salaam, Tanzania: A cross-sectional study. Int. J. Nephrol. **2020** (2020). 10.1155/2020/8378947 PMC7568141 33101732

[14] Peck, R. N. et al. Hypertension, kidney disease, HIV and antiretroviral therapy among Tanzanian adults: A cross-sectional study. *BMC Med.* **12**(1), 1–11 (2014). 10.1186/s12916-014-0125-2 PMC4243281 25070128

[15] Mwanjala, M. N., Urio, L. J. & Mtebe, M. V. Prevalence and predictors of renal dysfunction among people living with HIV on antiretroviral therapy in the southern highland of Tanzania: A hospital-based cross-sectional study. Pan Afr. Med. J. **41** (2022). 10.11604/pamj.2022.41.137.27025 PMC9034560 35519167

[16] Mapesi, H. et al. Prevalence, incidence and predictors of renal impairment in persons with HIV receiving protease-inhibitors in rural tanzania. *PLoS ONE* **16**(12), 0261367 (2021). 10.1371/journal.pone.0261367 PMC8673654 34910776

[17] Panga, O. D. et al. Prevalence, recent infection and predictors of HIV infection in fishing community along the shore of lake Victoria in Tanzania. *J. Public Health* **44**(4), 881–890 (2022). 10.1093/pubmed/fdab189 34117773

[18] Nsagha, D. S. et al. Haart, dots and renal disease of patients co-infected with HIV/AIDS and tb in the south west region of Cameroon. *BMC Public Health* **15**, 1–8 (2015). 26452646 10.1186/s12889-015-2331-z PMC4600262

[19] Peters, P. J. et al. Antiretroviral therapy improves renal function among HIV-infected Ugandans. *Kidney Int.* **74**(7), 925–929 (2008). 18614998 10.1038/ki.2008.305

[20] Calza, L. et al. Prevalence of chronic kidney disease among HIV-1-infected patients receiving a combination antiretroviral therapy. *Clin. Exp. Nephrol.* **23**, 1272–1279 (2019). 31327092 10.1007/s10157-019-01768-9

[21] Belay, A. S., Manaye, G. A., Kebede, K. M., Abateneh, D. D. & Debebe, S. Chronic kidney disease and its predictors among highly active antiretroviral therapy naïve and experienced HIV-infected individuals at the selected hospitals, southwest Ethiopia: A comparative cross-sectional study. BMJ Public Health **1**(1) (2023). 10.1136/bmjph-2023-000235 PMC11812716 40017843

[22] Mocroft, A. et al. Cumulative and current exposure to potentially nephrotoxic antiretrovirals and development of chronic kidney disease in HIV-positive individuals with a normal baseline estimated glomerular filtration rate: A prospective international cohort study. *Lancet HIV* **3**(1), 23–32 (2016). 10.1016/S2352-3018(15)00211-8 26762990

[23] Adedeji, T. A., Adebisi, S. A., Adedeji, N. O., Biliaminu, S. A. & Olanrewaju, T. O. Effects of highly active antiretroviral therapy on renal function and renal phosphate handling in African adults with advanced HIV and CKD. *Infect. Disord.-Drug Targets (Formerly Curr. Drug Targets-Infect. Disord.)* **19**(1), 88–100 (2019). 10.2174/1871526518666180720115240 30027856

[24] Nyende, L., Kalyesubula, R., Sekasanvu, E. & Byakika-Kibwika, P. Prevalence of renal dysfunction among HIV infected patients receiving tenofovir at Mulago: A cross-sectional study. *BMC Nephrol.* **21**, 1–6 (2020). 10.1186/s12882-020-01873-y PMC7310064 32571236

[25] Hull-Nye, D., Malik, B., Keshavamurthy, R. & Schwartz, E. J. Transient dynamics of the renal disease epidemic among HIV-infected individuals. *Math. Appl. Sci. Eng.* **1**(4), 373–382 (2020).

[26] Moya, E. M. D., Rodriguez, R. A. & Pietrus, A. A mathematical model for the study of HIV/AIDS transmission with PrEP coverage increase and parameter estimation using MCMC with a Bayesian approach. *Bull. Biomath.* **2**(2), 218–244 (2024).

[27] Luengo, D., Martino, L., Bugallo, M., Elvira, V. & Särkkä, S. A survey of Monte Carlo methods for parameter estimation. *EURASIP J. Adv. Signal Process.* **2020**, 1–62 (2020).

[28] Andrieu, C., De Freitas, N., Doucet, A. & Jordan, M. I. An introduction to MCMC for machine learning. *Mach. Learn.* **50**, 5–43 (2003).

[29] Moya, E. M. D., Rodrigues, D. S., Pietrus, A. & Severo, A. M. A mathematical model for HIV/AIDS under pre-exposure and post-exposure prophylaxis. *Biomath* **11**(2), 2208319–2208319 (2022).

[30] Amadi, M., Shcherbacheva, A. & Haario, H. Agent-based modelling of complex factors impacting malaria prevalence. *Malar. J.* **20**, 1–15 (2021). 33858432 10.1186/s12936-021-03721-2 PMC8048062

[31] Hernandez-Vargas, E. A. & Middleton, R. H. Modeling the three stages in HIV infection. *J. Theor. Biol.* **320**, 33–40 (2013). 23238280 10.1016/j.jtbi.2012.11.028

[32] Yoshimura, K. Current status of HIV/AIDS in the art era. *J. Infect. Chemother.* **23**(1), 12–16 (2017). 27825722 10.1016/j.jiac.2016.10.002

[33] Rivera, J. M. & Hashmi, M. HIV nephropathy. StatPearls (2023). 32644560

[34] Karacan, I., Sennaroglu, B. & Vayvay, O. Analysis of life expectancy across countries using a decision tree. *East. Mediterr. Health J.* **26**(2), 143–151 (2020). 32141591 10.26719/2020.26.2.143

[35] Marcus, J. L. et al. Narrowing the gap in life expectancy between HIV-infected and HIV-uninfected individuals with access to care. *J. Acquire. Immune Defic. Syndr.* **73**(1), 39 (2016). 10.1097/QAI.0000000000001014 PMC5427712 27028501

[36] Hethcote, H. W. The mathematics of infectious diseases. *SIAM Rev.* **42**(4), 599–653 (2000).

[37] Wairimu, J., Chirove, F., Ronoh, M. & Malonza, D. M. Modeling the effects of insecticides resistance on malaria vector control in endemic regions of Kenya. *Biosystems* **174**, 49–59 (2018). 30240719 10.1016/j.biosystems.2018.09.002

[38] Driessche, P. & Watmough, J. Reproduction numbers and sub-threshold endemic equilibria for compartmental models of disease transmission. *Math. Biosci.* **180**(1–2), 29–48 (2002). 12387915 10.1016/s0025-5564(02)00108-6

[39] Disease Control, C. & Prevention, U. Morbidity and Mortality Weekly Report. US Department of Health, Education, and Welfare, Public Health Service.

[40] UNAIDS: HIV estimates with uncertainty bounds 1990-Present. https://www.unaids.org/en/resources/documents/2023/HIV_estimates_with_uncertainty_bounds_1990-present. Accessed: 2023-09-30 (2023).

[41] Haario, H., Laine, M., Mira, A. & Saksman, E. Dram: Efficient adaptive MCMC. *Stat. Comput.* **16**, 339–354 (2006).

[42] UNAIDS: The urgency of now: AIDS at a crossroads. Joint United Nations Programme on HIV/AIDS Geneva (2024).

[43] World Health Organization: Country Cooperation Strategy: 2022-2027 - United Republic of Tanzania. https://www.afro.who.int/sites/default/files/2022-04/CCS%20Tanzania.pdf. Accessed: 2025-02-11 (2022).

[44] Crum, N. F. et al. Comparisons of causes of death and mortality rates among HIV-infected persons: Analysis of the pre-, early, and late HAART (highly active antiretroviral therapy) eras. *JAIDS J. Acquir. Immune Defic. Syndr.* **41**, 194–200 (2006). 16394852 10.1097/01.qai.0000179459.31562.16

[45] Nforbugwe, A. C., Asongalem, A. E., Nchotu, B. R., Tanue, E. A., Wirsiy, F. S. & Assob, N. J. Assessment of the effect of HAART on renal function of HIV patients attending the Bamenda regional hospital, Cameroon. Open AIDS J. **14**(1) (2020).

[46] Mahajan, V. K. et al. Assessment of liver and renal functions in human immunodeficiency virus-infected persons on highly active antiretroviral therapy: A mixed cohort study. *Indian J. Dermatol. Venereol. Leprol.* **86**, 499 (2020). 31975695 10.4103/ijdvl.IJDVL_169_18

[47] Chitnis, N., Hyman, J. M. & Cushing, J. M. Determining important parameters in the spread of malaria through the sensitivity analysis of a mathematical model. *Bull. Math. Biol.* **70**(5), 1272–1296 (2008). 18293044 10.1007/s11538-008-9299-0

[48] Omame, A., Han, Q., Iyaniwura, S. A., Ebenezer, A., Bragazzi, N. L., Wang, X., Kong, J. D. & Woldegerima, W. A. Understanding the impact of HIV on MPOX transmission in an MSM population: A mathematical modeling study. Infect. Dis. Modell. (2024). 10.1016/j.idm.2024.05.008 PMC11253271 39022298

[49] Msango, L. et al. Renal dysfunction among HIV-infected patients starting antiretroviral therapy in Mwanza, Tanzania. *AIDS* **25**(11), 1421 (2011). 21572304 10.1097/QAD.0b013e328348a4b1 PMC3631352

